# Advanced Green Extraction Methods for Valorising Artichoke Waste: Bioactive Composition, Stabilisation, and Implications for Nutrition and Disease Prevention

**DOI:** 10.3390/foods15122048

**Published:** 2026-06-06

**Authors:** Batuwitage Kosambi Hansini Hirupraba Batuwita, Andrew Tilley, Sung Tong Chin, Costas Stathopoulos

**Affiliations:** 1 School of Medical, Molecular, and Forensic Sciences, Murdoch University, Murdoch, WA 6150, Australia; hansini.batuwita@murdoch.edu.au (B.K.H.H.B.); andrew.tilley@murdoch.edu.au (A.T.); sung.chin@murdoch.edu.au (S.T.C.); 2 Food Futures Institute, Murdoch University, Nambeelup, WA 6150, Australia

**Keywords:** antioxidants, artichoke by products, deep-eutectic solvents, encapsulation, flavonoids, health benefits, nutritional properties, polyphenols, sustainability

## Abstract

Globe artichoke (*Cynara scolymus*) processing generates large amounts of agro-industrial waste, including stems, leaves, and bracts. These by-products represent a valuable and underexplored source of bioactive compounds, particularly phenolic acids, flavonoids, pectin, and inulin, which exhibit significant nutritional, health-promoting, and functional properties. This review provides a comprehensive overview of green extraction strategies applied to the recovery of bioactive compounds from globe artichoke waste, with emphasis on green extraction techniques such as deep eutectic solvent, ultrasound-assisted, microwave-assisted, enzyme-assisted, subcritical water, supercritical CO_2_, and the use of green solvents. Nutritional composition and biological activities, including antioxidant, antimicrobial, anti-inflammatory, hypolipidemic, and hepatoprotective effects of artichoke waste extracts, are critically discussed. Given the inherent instability and limited bioavailability of many phenolic compounds, recent advances in encapsulation and stabilisation approaches, alginate-based systems, spray-drying, and nano and microencapsulation technologies are highlighted as effective strategies to enhance shelf life and controlled release. The valorisation of globe artichoke waste through green extraction and encapsulation of bioactive compounds contributes to circular economy principles by reducing environmental impact while adding value. Overall, the promising role of artichoke by-products as sustainable resources for functional food development is discussed.

## 1. Introduction

**Figure 1:**
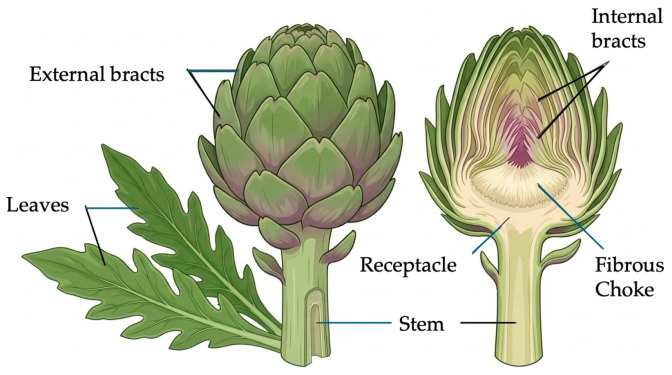
Cross-section of globe artichoke inflorescence (created using FigureLabs and PowerPoint).

Globe artichoke (*Cynara cardunculus*) is a perennial herb native to the Mediterranean region and is cultivated in countries such as Egypt, Italy, Spain, Algeria, Peru, and China due to the crop’s adaptability to diverse soil types and climatic conditions [1,2,3]. Artichoke plants are distinguished by their deeply lobed green leaves and flower heads known as inflorescence made up of green to violet fleshy bracts that encircle the receptacle, commonly referred to as the heart [1,3]. The edible portion (EP) of the artichoke inflorescence is approximately 43% of the whole inflorescence. It is composed of the receptacle, the inner bracts and the inner section of the stem; the remaining 57% consists of the outer bracts, choke, and stalk [4] (Figure 1). The EP is widely consumed in many forms including raw, boiled, roasted, baked, canned or fried [1,2].

During artichoke harvesting and replacing the crops, the leaves, external bracts, and a large portion of the stems are removed, producing substantial waste that is usually discarded in the field. This waste accounts for about 70–85% of plant biomass [1,4,5]. Industrial processing for canned artichokes also generates a significant amount of artichoke waste bracts, stems, leaves, and by-products, which are generally limited to low-value applications such as animal feed [3,4], despite containing high-value bioactive compounds such as inulin, vitamins, essential minerals, sesquiterpene lactones, inositol, and polyphenolic compounds [1]. This review evaluated the effective application of green extraction methods for obtaining bioactive compounds from artichoke waste, with emphasis on their composition, bioactivity, nutritional value, bio accessibility, and disease-prevention potential. The stabilisation of extracted phenolics through encapsulation was discussed, highlighting novel and future directions of the sustainable utilisation of artichoke waste.

Existing studies have predominantly focused on the extraction and identification of phenolic compounds from artichoke by-products, with primary emphasis on optimising extraction yield and antioxidant activity. However, there is limited interpretation of evidence regarding the comparative effectiveness of the extraction methods, the stabilisation of extracted phenolics and their food applications.

Although the biological activities of artichoke-derived phenolics have been widely reported, relatively only a few studies have critically examined their bioaccessibility, bioavailability, gastrointestinal stability, and metabolic transformation following ingestion. As a result, the relationship between extracted phenolic composition and physiological efficacy remains poorly understood.

Current literature also lacks a comprehensive evaluation of stabilisation strategies, such as encapsulation and carrier-based delivery systems, that could improve the storage stability and controlled intestinal release of these compounds. There remains a need for a comprehensive review that integrates green extraction methods (GEMs), compound stabilisation, bioavailability of the compounds, and their health implications within a sustainable framework.

This review aims to critically evaluate advanced GEMs for the sustainable valorisation of artichoke waste, with particular emphasis on their efficiency, environmental sustainability, and ability to preserve the bioactivity of phenolic compounds. The review further seeks to examine the bioactive composition, bioaccessibility, bioavailability, and stabilisation of artichoke-derived phenolics, and to assess their implications for nutrition and disease prevention. In addition, the commercial and industrial applicability of artichoke phenolics as functional food ingredients or nutraceuticals are also evaluated, while identifying current challenges, research limitations, and future directions are necessary for large-scale sustainable utilisation.

The literature survey for the published articles was done from September 2025 to May 2026, mainly focusing on English-language, peer-reviewed research articles published between 2020 and 2026. Following the first round of reviews, a few articles from the years 2000–2020 were included to facilitate addressing the reviewers’ comments. The keywords such as “artichoke by products”, “green extraction methods”, “artichoke phenolic compounds”, “encapsulation methods”, “bioavailability and bioaccessibility of phenolic compounds”, and “food applications” were used to search published research articles available in scientific databases including Google Scholar, Scopus, PubMed, CrossRef and Web of Science. Since the review focused on phenolic compounds, studies carried out on horticultural and agricultural practices, genetics of the artichoke plant, without addressing byproduct conversion, and other bioactive compounds in artichoke by-products were excluded. Additionally, the studies that fail to provide complete quantitative data, clear extraction parameters (e.g., temperature, solvent-to-solid ratio), or sufficient statistical validation were also not considered in this review.

## 2. Bioactive Compounds in Artichoke Waste

Artichoke waste is a rich source of various phenolic compounds, known for their diverse health-promoting properties and their role in plant defence and adaptation [6]. Hydroxycinnamic acids, such as cis and trans 3-O-caffeoylquinic acids and flavonoids including flavones (luteolin glycoside derivatives) are the principal phenolic constituents of artichoke waste [7]. The predominant phenolic compounds in artichoke leaves in several genotypes were flavonoids (57–78%) [7,8]. The total phenolic content (TPC) and the phenolic profile vary between plant parts, and according to [9], artichoke head waste (1743–8361 mg gallic acid equivalent (GAE) kg^−1^ fresh weight (fw)) had the highest TPC, followed by leaf (1411–6447 mg GAE kg^−1^ fw), head (1845–6435 mg GAE kg^−1^ fw), and stem (1459–6163 mg GAE kg^−1^ fw), respectively, highlighting the variance in TPC based on the type of waste. The TPC was highest in the receptacle (heart) (48.9 mg g^−1^ dry matter (DM)) and stem (45.7 mg g^−1^ DM), while bracts showed significantly lower values (27.4 mg g^−1^ DM) [10]. A similar pattern was observed for chlorogenic acid (CGA) content, with higher values in the heart (24.2 mg g^−1^ DM) and stem (21.4 mg g^−1^ DM) than in bracts (5.8 mg g^−1^ DM), indicating greater antioxidant potential in the edible fractions. The difference in the TPC reported in different studies may be due to significant variations in the cultivar, genotype, fertilisers used, climatic conditions, other inherent factors, and the method of extraction.

### 2.1. Nutritional Benefits and Disease Prevention Implications of Phenolics Extracted from Artichoke Waste

Polyphenols are widely recognised for their health-promoting antioxidant, anti-inflammatory, antimicrobial and chemopreventive effects [11]. The antioxidant capacity of plant extracts is closely associated with their potential clinical use in the management of gastrointestinal disorders related to oxidative stress [12]. Dietary phenolic compounds are linked to reduced risk of chronic and degenerative diseases associated with oxidative stress, as polyphenols can counteract elevated levels of reactive oxygen species (ROS) implicated in disorders such as inflammatory bowel disease and cancer [12]. Cynaropicrin is a key representative compound in artichoke, and the compound’s guaianolide-type sesquiterpene lactone is recognised as a chemotaxonomic marker and contributes nearly 80% of the typical bitter taste by activating bitter sensory receptors [11]. Cynaropicrin has potential protective effects against photoaging and cosmetic disorders by suppressing ROS generation and reducing the production of inflammatory cytokines in ultraviolet B–irradiated keratinocytes [13]. Moreover, cynaropicrin exhibits significant anti-hepatitis C virus, antihyperlipidemic, anti-inflammatory, and antitumoral activities [14,15].

Artichoke consists of proven antioxidant, anticancer and anti-inflammatory properties [11]. Artichoke waste extracts contain 5-O-caffeoylquinic acid (CGA) and 3,4-di-Ocaffeoylquinic acid as the most prevalent hydroxicinnamates, while apigenin-7-O-rutinoside and luteolin-7-O-glucoside are the major flavonoids found in all analysed genotypes [1], and these extracts can be effective as a prebiotic in increasing the cell density of different probiotics. However, the actual prebiotic potential still requires targeted evaluation, especially since earlier studies have reported contradictory results claiming that artichoke extracts display antimicrobial activity against both Gram-positive and Gram-negative bacteria [12,13].

CGA serves as an important quality control marker in the trade of artichoke-based herbal materials. According to the European Pharmacopoeia (Ph. Eur.), artichoke leaves intended for herbal drug use must contain a minimum of 0.7% CGA [1], and their study showed that CGA was the most abundant polyphenol, with 24.9 µg mg^−1^ of concentration.

Hydrogen peroxide exposure is widely used to simulate the pro-oxidative conditions characteristic of degenerative diseases, such as cancer and neurodegenerative disorders, in 2D cell culture models. The extracted hydroxycinnamic acids from artichoke waste on Caco-2 cells (in vitro) showed that the antioxidant effect was dependent on both exposure time and the concentration [14]. Hydroxycinnamic acids have considerable potential in managing oxidative stress in the gastrointestinal tract. In Jimenez-Moreno et al. [14], all tested concentrations of extracts (250–1000 µg mL^−1^) had significantly reduced hydrogen peroxide-induced ROS production, with 1000 µg mL^−1^ being the most effective, indicating the significance of the extract concentration in determining antioxidant capacity.

ROS are natural anaerobic metabolic by-products, but oxidative stress arises when pro-oxidants overwhelm antioxidant defences. This state is often linked to inflammation and it is a major factor in the development of chronic diseases like non-alcoholic fatty liver disease (liver steatosis) and nonalcoholic steatohepatitis [15,16,17]. Acquaviva et al. [15] studied how phenolics from artichoke leaves could alleviate oxidative stress conditions using an in vitro simulation of early-stage liver steatosis. Notably, at a concentration of 50 µg mL^−1^, the extract restored cell viability to levels indistinguishable from those of the control group, presenting its significant potential influence on liver health. A 12 h exposure to free fatty acids (FFAs) significantly elevated lipid hydroperoxide (LOOH) levels in HepG2 cells, demonstrating a protective effect only at the 50 µg mL^−1^. Moreover, artichoke phenolics can protect the hepatocytes from oxidative stress by inducing apoptosis in human hepatoma and human breast cancer cell lines, while showing no toxicity toward nontumorigenic MCF10A cells, thereby exhibiting chemopreventive anticancer properties [18].

Artichoke polyphenols have demonstrated a strong ability to regulate starch digestion, thereby contributing to potential antidiabetic activity [19]. In this context, artichoke exhibits anti-obesity properties, which are linked to the possible inhibition of starch-digesting enzymes [20]. In the in vivo study of Mejri et al. [21], the administration of the artichoke floral stem (AFS) extract restored serum biochemical parameters and rebalanced the endogenous antioxidant defence system against free radicals. This was evidenced by increased activities of catalase (CAT), glutathione peroxidase (GPx), and superoxide dismutase (SOD, Cu/Zn-SOD, Fe-SOD, and Mn-SOD), along with decreased lipid peroxidation and reduced H_2_O_2_ levels in both hepatic and renal tissues. Collectively, these findings suggest that AFS extract alleviated diabetic conditions, likely by promoting insulin production and/or secretion, improving cellular insulin sensitivity, and enhancing glucose tolerance. AFS extract has the potential to reduce the lipid levels and protect against the onset of atherosclerosis and cardiovascular complications in diabetic animals. The lipid-lowering effect of luteolin was attributed to its suppression of inflammatory pathways, whereas the cholesterol-lowering activity is associated with the inhibition of cholesterol biosynthesis [22]. Although the precise mechanisms remain unclear, the hypolipidemic effect of AFS extract is likely related to its ability to stimulate insulin secretion and to suppress hormone-sensitive lipase activity in adipose tissue through insulin action. Therefore, further research should be focused on identifying the responsible phenolic compounds, their effective concentrations, and the interactions that support the actual mechanism. This will lead to study on optimum extraction, stabilisation methods, and storage conditions more specifically to the targeted compound.

Phenolic compounds, particularly CGA, are believed to exert neuroprotective effects by counteracting oxidative and excitotoxic stress in the central nervous system, and pathological conditions that are implicated in the development of neurodegenerative diseases such as Alzheimer’s, Parkinson’s, and Huntington’s [23,24,25]. The in vitro study of Gao et al. [26] showed the neuroprotective effect of CGA on human neuroblastoma SHSY5Y cells damaged by H_2_O_2_, a model commonly used to examine oxidative stress-induced neuronal cell death and to evaluate the neuroprotective potential of compounds. Interestingly, CGA could reduce the observed morphological changes and lower the cell apoptosis in a manner dependent on both dose and exposure time. CGA can mitigate the oxidative stress and its associated detrimental effects by helping to maintain a balanced intracellular redox state [6]. The antioxidant and anti-inflammatory properties of artichoke waste cannot be ascribed solely to CGA; instead, it arises from the combined presence of multiple essential phenolic compounds, highlighting the synergistic effects involved [6]. Given that significant effects were observed at 25 µg mL^−1^ of the extract in in vitro analysis, it is essential to determine whether comparable concentrations can be attained in vivo. Olthof et al. [27] showed that 33% of the initial intake of CGA was absorbed in human gut, while caffeic acid showed higher absorption with 95% of the initial intake. Future studies should focus on elucidating the bioavailability and pharmacokinetic profiles of the extract and individual phenolic compounds in vivo and, ultimately, in humans after their oral administration [6]. In Brahmi-Chendouh et al. [28] with in vitro Caco-2 cells, phenolics from the stem exhibited greater effectiveness (>70% at around 170 µg mL^−1^) in redox activity inhibition (RAI), whereas the leaves and capitula displayed nearly overlapping dose–response curves, with the capitula achieving a maximum RAI of 40.2% only at the highest concentration tested (200 µg mL^−1^). AFS extract was also successful in suppressing heat-induced albumin denaturation, indicating a potential in vitro anti-inflammatory effect [21]. This activity showed a strong positive correlation with TPC, total flavonoid content (TFC) (r = 0.889), and the DPPH radical scavenging activity (r = 0.963), implying that phenolic constituents in the extract are the primary contributors to the observed in vitro anti-inflammatory activity. Similar relationships between DPPH scavenging activity (IC_50_ value) and the anti-inflammatory properties were confirmed in previous studies on plant extracts [29,30]. In Carpentieri et al. [31], using an in vitro model of cellular oxidative stress and inflammation using lipopolysaccharides stimulated human THP-1 macrophages, artichoke extract was found to reduce ROS production, as well as the gene expression and protein secretion of Interleukin-6 and chemokine ligand 2. These findings suggest that DPPH radical scavenging capacity can serve as a reliable predictor of the in vitro anti-inflammatory potential of artichoke floral stem extracts, further confirming the close association between antioxidant and anti-inflammatory activities.

Phenolics commonly contribute to antimicrobial properties and function as a chemical defence mechanism that protects against microbial invasion [32]. AFS showed an apparent antimicrobial activity against a set of six microorganisms and found that *S. aureus* and *E. faecium* were the most susceptible strains, exhibiting the largest zones of inhibition [21]. In Turksever et al. [32], when treated with artichoke leaf extract, the antibacterial efficacy was higher toward Gram-positive bacteria (*Staphylococcus aureus*, *Bacillus cereus*, *Bacillus subtilis* ssp. *spizizenii*, *Enterococcus faecalis*, and *Listeria monocytogenes*), while spore-forming microorganisms exhibited lower susceptibility. Identification of the specific phenolic compounds and effective doses responsible for strain-specific antimicrobial activity is necessary to accurately evaluate their therapeutic potential. Further investigations should focus on the functional effects of individual bioactive compounds, alongside dose–response analyses and long-term safety assessments to establish optimal efficacy and potential toxicity profiles.

### 2.2. Bioaccessibility, Bioavailability and Metabolism of Polyphenols Extracted from Artichoke Waste

The use of artichoke extracts as functional food supplements requires evaluation of their safety, compositional attributes related to health effects, and their stability during digestion, which may impact their bioaccessibility [28]. Both bioactivity and cellular absorption are closely linked to chemical structure. For phenolics to exert their beneficial effects, they need to be released during digestion, emphasising the critical role of the food matrix in determining their bioaccessibility [33]. The bioaccessibility of phenolics is governed by several variables, such as their molecular characteristics, the surrounding food matrix, interactions with other compounds, and the presence of suppressing agents or cofactors [34].

In Kayahan et al. [33], carried out with simulated in vitro digestion, cooking (boiling, microwave cooking and baking) could markedly improve the bioaccessibility of specific phenolic compounds and influence the antioxidant characteristics of artichoke hearts (different genotypes) throughout digestion. Cooking methods such as microwaving and boiling generally enhanced TPC and CUPRAC antioxidant bioaccessibility (especially during the intestinal phase), and it can be assumed that the cell rupturing caused by high temperature or microwave processing has led to increased release of heat-stable phenolics such as cynarine. Meanwhile, heat-sensitive phenolic compounds such as syringin (a glucoside) and luteolin (a flavonoid) are susceptible to thermal degradation during cooking. These compounds are characterised by linkages to other organic moieties through glycosidic, C–C, and C–O–C bonds. Exposure to heat, particularly in the presence of water (hydrolytic conditions), facilitates the cleavage of these bonds, leading to the breakdown of phenolic structures. As a result, the molecules dissociate into constituents such as glucose, gallic acid, caffeic acid, aromatic components, and aglycones [33]. Syringin, being a phenolic glycoside, gets glycosidic bonds broken due to gastric acidity and enzymatic activity in the small intestine, leading to the disruption of its original molecular structure [33]. The cooking or heat processing method chosen significantly influences the bioaccessibility of phenolic compounds. The choice of cooking method and the optimal process conditions should be applied based on the targeted bioactive compounds to be preserved and the artichoke genotype used.

Phenolic compounds from capitula, stems, and leaves in natural deep eutectic solvent (NaDES) based food preparations are predominantly released during the gastric phase, while among the flavonoids, luteolin and apigenin derivatives originating from the capitula show a positive association with the composition of the final digesta [28]. Monocaffeoylquinic acids from artichoke increase under gastric conditions, while the shift to the intestinal phase leads to a marked rise in overall phenolics recovery. In both in vitro studies of Brahmi-Chendouh et al. [28] and D’Antuono et al. [35], an increase in artichoke monocaffeoylquinic acids was observed under gastric conditions, while exposure to intestinal conditions resulted in a significant rise in recovery of total phenolics. Alterations in the chemical composition of ingested foods are closely associated with digestive processes, which modify the structures of bioactive compounds while either preserving or altering their biological activity [36]. CGAs and syringin in artichoke heads are found to be potentially susceptible to conditions in the gastric and small intestinal environments. However, in Brahmi-Chendouh et al. [28], capitula-based samples had maintained their efficacy following both the gastric and intestinal phases, likely because of the release of apigenin and luteolin glycosides in addition to 1,3-dicaffeoylquinic acid. Dose-dependent effects were observed, with capitula formulations (73.9 and 62.4%) prior to simulated digestion showing the highest scavenging capacity toward both DPPH and ABTS radicals, followed by leaf (49.1 and 57.7%) and stem (26.5 and 29.2%) [28]. Alongside the breakdown of polyphenolic structures, certain isomerisation reactions occur during the gastrointestinal digestion of 5-O-caffeoylquinic acid (5-CQA). In particular, the conversion of 5-CQA to 3-CQA is strongly influenced by pH and is likely to occur during the intestinal phase of digestion (pH 7) [33]. A study in humans (clinical analysis) showed that the disposition of caffeoylquinic acid metabolites in plasma is dose-dependent [37]. In another human trial, while some phenolics (3-CQA and 4-CQA) entered the plasma in an unmetabolized state, they were rapidly and extensively 3′-methylated in plasma [38]. Wittemer et al. [37] also reported similar findings that after entering the systemic circulation, CQA conjugates were methylated during the first pass through the liver. However, this methylation activity can be obscured when beverages like coffee and fruits (apple, berry, peach, etc.) are consumed due to the presence of pre-existing phenolics within them [38,39].

Exposure to acidic conditions often leads to a noticeable increase in TPC and enhanced antioxidant activity, driven by several underlying mechanisms. The phenolic compounds, typically enclosed within plant cell walls, composed of cellulose, hemicellulose, and pectin, can be easily released under acidic conditions, particularly when combined with mild heat (e.g., during cooking), and partial hydrolysis [33]. The degradation of these structural polysaccharides occurs, facilitating the release of previously bound phenolics and thereby elevating the TPC. Additionally, many phenolic compounds are present as glycosides or esters; thereby, acidic environments promote the cleavage of these linkages, releasing free phenolic forms (aglycones) that are more readily detectable and possess greater antioxidant potential and bioaccessibility after the heat process [33].

It is important to highlight pharmacokinetic limitations and bioavailability issues that arose in various studies. In Wittemer et al. [37], CQAs were detected in human plasma and urine as conjugated and unconjugated hydroxycinnamates. Nevertheless, the conjugated forms, such as sulfates or glucuronides, could not be specifically identified in this study. This limitation was likely due to the analytical methods being designed mainly for the parent compounds, causing the conjugates to coelute with other matrix components. In human clinical studies, the bioactivity of consumed phenolics is influenced by both their metabolites and the concentrations attained within the body. However, considerable interindividual differences exist in the metabolic profiles of these compounds (between 3′- and 4′-methylation of 3′,4′-dihydroxyphenyl-substituted substrates) [38]. Consequently, there is growing interest in understanding how such variability affects the bioavailability and metabolism of phenolics in order to better explain their beneficial effects [40].

## 3. Advanced Green Extraction Methods (GEMs) to Extract Polyphenols from Artichoke Waste

The extraction method used for recovering phenolic compounds plays a critical role in determining their composition, stability, and bioavailability; different extraction techniques can influence the yield, structural integrity, and release of phenolics by affecting factors such as solvent polarity, temperature, and extraction time. Efficient extraction methods help preserve bioactive compounds and enhance their accessibility for absorption in the human body, thereby improving their functional and health-promoting properties. Therefore, selecting an appropriate extraction approach is essential for maximising the bioavailability and overall efficacy of phenolic compounds in food and nutraceutical applications.

Conventional extraction methods commonly rely on solid–liquid extraction (SLE) using volatile organic solvents such as ethanol, methanol, or acetone as extractants; however, these approaches may lead to the retention of undesirable solvent residues in the final extract [41,42]. These solvents are increasingly discouraged in current research due to concerns related to their toxicity, high volatility, and non-renewable nature (acetone, diethyl ether, n-hexane, toluene, etc.), which pose environmental and safety challenges. Their application is also tightly controlled under international regulations, including Registration, Evaluation, Authorisation and Restriction of Chemicals (REACH) presented by the European Union [41]. These conventional methods frequently involve high temperatures and extended extraction time, leading to considerable energy usage [2]. Accordingly, there is an increasing demand for environmentally friendly alternative solvents and GEMs (refer to Table 1) that minimise ecological impact while enhancing downstream processing efficiency [41]. GEMs increase operator safety, reducing energy consumption and improving waste management, while replacing hazardous chemicals with environmentally friendly alternatives (water and food-grade organic acids) whenever possible [43]. Following the principles of green chemistry, it is crucial to substitute conventional petroleum-based solvents with environmentally friendly alternatives to promote sustainable downstream processing [44,45].

Bioactive compounds can be effectively extracted from different plant tissues through SLE using solvents with suitable polarity. Advanced GEMs, including deep-eutectic solvent extraction (DESE), high-pressure assisted extraction (HPAE), subcritical water extraction (SWE), ultrasound-assisted extraction (UAE), pulsed electric field extraction (PEFE), enzyme-assisted extraction (EAE), Supercritical carbon dioxide extraction (SCO_2_E), and microwave-assisted extraction (MAE), though more sophisticated, provide several advantages such as enhanced stability of polyphenols, minimal or no solvent usage, higher extraction efficiency, and lower energy requirements [1,46].

**Table 1:** Different extraction methods, TPC and antioxidant activity, phenol profile of artichoke waste products, and the yield, advantages and limitations of the extraction methods.

Extraction Method	TPC	TFC	Antioxidant Activity	Phenolic Profile	Yield	Important Findings	Energy Consumption, the Environmental Impact, Advantages and Limitations	Reference
Artichoke leaves								
Hot water extraction at 90 °C for 1 h (solid: solvent = 1:10)	185.21 mg GAE g^−1^ extract	50.32 mg CEg^−1^ extract	DPPH–IC_50_ 20.04 µg mL^−1^ (15 µM TE)	ND	5.6 g of dry extract (d.e) 100 g fw ^−1^	MTT assay-Treatment for 24 h of the extract (10–50 µg mL^−1^) was not cytotoxic for HepG2 cells HepG2 cells exposed for 12 h to FFAs (1.5 mM oleic acid and palmitic acid (2:1)) at a concentration of 1.5 mM showed a significant increase in ROS and LOOH levels, and reduced viability (40%) Pre-treatment with the crude extract (50 µg mL^−1^) was effective in reducing ROS production	Simple and less costly process High energy consumption due to heating at 90 °C for prolonged period It is hard to remove the solvent; therefore, recovery needs extra steps through evaporation Since water is used as the extracting solvent, this method is environmentally friendly, yet high heat consumption reduces the benefit	[15]
UAE (EtOH: water (50:50, *v*/*v*, amplitude and impulse = 100) with sonotrode	7.22 mg g^−1^ dp 215.08 mg GAE g^−1^ dp Total hydroxycinnamic acids content (THC)-1.18 mgg^−1^ dp Total hydroxybenzoic acid content (THB)-1.06 mgg^−1^ dp	4.98 mgg^−1^ dp	CUPRAC-3.79 mmol Fe^2+^ g^−1^ dp DPPH-0.52 mmol TEAC g^−1^ dp ABTS-1.20 mmol TEAC g^−1^ dp FRAP-2.35 mmol Fe^2+^ g^−1^ dp	Flavonoid Luteolin 7-O-rutinoside–970 µg g^−1^ dp Luteolin 7-O-glucoside–1530 µg g^−1^ dp Apigenin rutinoside–350 µg g^−1^ dp Apigenin glucuronide–230 µg g^−1^ dp Luteolin–250 µg g^−1^ dp Other flavonoids-1640 µg g^−1^ dp Hydroxycynnamic acids CGA–630 µg g^−1^ dp Coumaroyl-quinic acid–100 µg g^−1^ dp Di-caffeoylquinic acid I–20 µg g^−1^ dp Di-caffeoylquinic acid II–30 µg g^−1^ dp Other hydroxycinnamic acids–410 µg g^−1^ dp	TPC yield-7.22 mg g^−1^ dp	The type of solvent was the most decisive extraction parameter for the performance (linear or quadratic terms) and the targeted compounds Pure ethanol reduced the extraction efficiency due to the hydrophilic nature of phenolics, while pure ethanol positively affects cynaropicrin, a weakly polar compound. Amplitude and impulse did not show significant influence on the target compound extraction	Simple and less costly process Energy can be saved due to extraction at reduced temperature Mild treatment of ultrasounds does not produce any significant changes in the properties and functionality of most of the bioactive compounds, which makes it ideal for the extraction of antioxidants UAE is a GEM; however, ethanol usage as an extracting solvent reduces its weight as a GEM Recovery of the solvents should be focused through rotary evaporation and condensation	[47,48]
Supercritical CO_2_ extraction (SCO_2_E) (40 °C, 300 bar)	1.53 mgg^−1^ dp 109.21 mgGAEg^−1^ dp THC–0.08 mgg^−1^ dp THB-ND		CUPRAC–5.88 mmol Fe^2+^ g^−1^ dp FRAP–0.93 mmol Fe^2+^ g^−1^ dp DPPH–0.10 mmol TEACg^−1^ dp ABTS–0.29 mmol TEACg^−1^ dp	Cynaropicrin-48.33 mgg^−1^ dp CynaroscolosideA/B-8.22 mg g^−1^ dp Luteolin 7-O-rutinoside-0.74 mg g^−1^ dp Luteolin 7-O-glucoside-0.78 mg g^−1^ dp	TPC yield–109.21 mg g^−1^ dp	Ideal for extracting and isolating weakly polar or non-polar compounds, and not suitable for extracting polar phenolics Due to weakly polar nature, cynaropicrin can efficiently be extracted using SCO_2_E SCO_2_E is a flexible extraction technique in which temperature and pressure can be modified to change the density of CO_2_, thereby controlling solubility.	Complex and costly process Energy can be saved due to extraction at reduced temperature High pressure combined with reduced temperature is eco-friendly SCO_2_E is a GEM that produces solvent-free extracts, causing minimal changes to bioactive compounds SCO_2_E offers a high level of selectivity during extraction SCO_2_E is mainly applied for extracting valuable non-polar to moderately polar compounds, such as essential oils. To improve the extraction of highly polar compounds, modifiers or cosolvents may be introduced, as SCO_2_E has limited efficiency for such molecules	
Subcritical Water Extraction (SWE) (EtOH: H_2_O (50:50, *v*/*v*), 125 °C)	23.39 mgg^−1^ dp 384.16 mgGAEg^−1^ dp THC-4.26 mgg^−1^ dp THB-2.77 mgg^−1^ dp	16.36 mgg^−1^ dp	CUPRAC–10.72 mmol Fe^2+^ g^−1^ dp FRAP–6.28 mmol Fe^2+^ g^−1^ dp DPPH–1.84 mmol TEACg^−1^ dp ABTS–1.93 mmol TEACg^−1^ d	Luteolin 7-O-glucoside-7.50 mgg^−1^ dp Luteolin 7-O-rutinoside-3.49 mgg^−1^ dp Chlorogenic acid-3.28 mgg^−1^ dp Cynaropicrin-5.41 mgg^−1^ dp	TPC yield-23.39 mgg^−1^ dp	With increasing temperatures, the levels of all phenolic compounds were reduced. Therefore, SWE is ideal for heat non labile compound extraction.	SWE can be ideal for extracting high-temperature-resistant compounds in high yield, yet it is unsuitable for preserving overall bioactivity Simple and less costly process High energy consumption due to heating at >100 °C for prolonged period To remove the solvent, evaporation is required This method is environmentally friendly, yet high heat consumption reduces the benefit High water consumption due to condensing	[47]
DES (Choline chloride: levulinic acid 1:2-H_2_O (80:20, *v*/*v*))	9.69 mgg^−1^ dp 123.03 mgGAEg^−1^ dp THB–1.67 mgg^−1^dp	ND	CUPRAC–1.96 mmol Fe^2+^g^−1^ dp FRAP–2.17 mmol Fe^2+^g^−1^ dp DPPH–0.38 mmol TEACg^−1^ dp ABTS–0.75 mmol TEACg^−1^ dp	Cynaropicrin-3.19 mgg^−1^ dp Luteolin 7-O-rutinoside–1.24 mgg^−1^ dp Luteolin 7-O-glucoside–1.84 mgg^−1^ dp Apigenin rutinoside–0.54 mgg^−1^ dp Chlorogenic acid–0.54 mgg^−1^ dp	TPC yield-9.69 mgg^−1^ dp	The extraction yield of cynaropicrin increases as the amount of hydrogen bond acceptor (HBA) decreases	Simple and less costly process Less energy consumption and environmentally friendly Since DESs have low volatility, it is difficult to remove the solvent from the extracts obtained and thereby the yield for downstream processes may be limited. To eliminate this drawback, phenolics could be adsorbed by resins, and followed by the elution with an organic solvent	
Microwave-assisted extraction (MAE) (800 W, 30 s, 50 °C) using a household microwave device. ETOH: water = 1:2 and solvent: solid = 10:1	3.72 GAE mgg^−1^ d.w.	8.30 CE mgg^−1^ d.w.	TEAC–57.9 TE mM g^−1^ d.w. DPPH–85.2%	Following phenolics were present CGA Luteolin Luteolin 4′-O-glucoside Luteolin 7-O-glucuronide Quercetin 3,7-dirhamnoside (polyphenol) 1,3-Di-O-caffeoylquinic acid (cynarine) 4,5-Di-O-caffeoylquinic acid 1-O-caffeoylquinic acid	ND	MAE > UAE with probe > UAE 30 min water bath > maceration > UAE 15 min water bath In terms of TPC The fast interaction between microwave energy and solvent molecules promotes rapid breakdown of cell walls, facilitating the release of bioactive compounds. Exposure time is a key factor in improving the efficiency of MAE. However, prolonged exposure to high microwave power may lead to the degradation of certain phenolic compounds. Extracts obtained through MAE (800 W) demonstrated strong effectiveness against a wide range of opportunistic and pathogenic microorganisms.	UAE, MAE and maceration are simple and less costly processes Energy can be saved due to extraction at reduced temperature Mild treatment of conditions in UAE does not produce any significant changes in the properties and functionality of most of the bioactive compounds, making it ideal for the extraction of antioxidants To remove the solvent, evaporation is needed MAE and UAE are GEMs; however, ethanol usage as an extracting solvent reduces their weight as a GEM Maceration for prolonged time (12–24 h) is a drawback	[32,49]
MAE (440 W, 45 s, 50 °C) using a household microwave device. ETOH: water = 1:2 and solvent: solid = 10:1	3.40 GAE mgg^−1^ d.w.	6.50 CE mgg^−1^ d.w.	TEAC–50.0 TE mM g^−1^ d.w. DPPH–70.7%	ND				
UAE with a probe (50 °C, (50% circle) and a continuous process (100% circles) in two different time periods of 15 or 30 min)	Continuous (30 min)-2.72 GAE mgg^−1^ d.w.	6.6 CE mgg^−1^ d.w.	TEAC–51.4 TE mM g^−1^ d.w. DPPH–84.1%					
	Continuous (15 min)-2.4 GAE mgg^−1^d.w.	6.1 CE mgg^−1^ d.w.	TEAC–42.2 TE mM g^−1^ d.w. DPPH–79.9%					
	Pulsed (30 min)-2.7 GAE mgg^−1^d.w.	6.5 CE mgg^−1^ d.w.	TEAC–52.0 TE mM g^−1^ d.w. DPPH–81.2%					
	Pulsed (15 min)-2.48 GAE mgg^−1^ d.w.	5.6 CE mgg^−1^ d.w.	TEAC–46.2 TE mM g^−1^ d.w. DPPH–77.4%					
UAE with a water bath (50 °C, in two different time periods of 15 or 30 min with two power values of 120 and 240 W)	240 W, 30 min–2.5 GAE mgg^−1^ d.w.	5.3 CE mgg^−1^ d.w.	TEAC–42.8 TE mM g^−1^ d.w. DPPH–69.4%					
	240 W, 15 min–1.9 GAE mgg^−1^ d.w.	3.2 CE mgg^−1^ d.w.	TEAC–32.4 TE mM g^−1^ d.w. DPPH–66.4%					
	120 W, 30 min-2.3 GAE mgg^−1^ d.w.	4.3 CE mgg^−1^ d.w.	TEAC–32.1 TE mM g^−1^ d.w. DPPH–69.9%					
	120 W, 15 min-1.9 GAE mgg^−1^ d.w.	4.0 CE mgg^−1^ d.w.	TEAC–34.0 TE mM g^−1^ d.w. DPPH–63.6%					
Maceration (50 °C for 12 and 24 h)	24 h–2.00 GAE mgg^−1^ d.w.	24 h–4.00 CE mgg^−1^ d.w.	TEAC–43.30 TE mM g^−1^ d.w. DPPH–69.40%					
	12 h–1.89 GAE mgg^−1^ d.w.	12 h–3.55 CE mgg^−1^ d.w.	TEAC–42.60 TE mM g^−1^ d.w. DPPH–60.0%					
Maceration (80% ETOH/water solution (*v*/*v*) at room temperature, 2 h)	169.4 μgGAEmg^−1^ Total CGA-32.1 μgCGAE	65.0 μg QE mg^−1^	DPPH IC_50_-0.14 mgmL^−1^ ABTS IC_50_-0.07 mgmL^−1^	**Hydroxycinnamic acids** CGA (5-O-caffeoylquinic acid (5-CQA))-24.9 μg mg^−1^ Caffeic acid-0.86 μg mg^−1^ 1,5-dicaffeoylquinic acid-6.00 μg mg^−1^ **Flavonoids** Luteolin-7-O-glucoside-14.5 μg mg^−1^ Luteolin-7-O-rutinoside-20.8 μg mg^−1^ Narirutin-4.73 μg mg^−1^ **Minor polyphenols** Tyrosol-0.05 μg mg^−1^ Syringic acid–0.15 μg mg^−1^ p-Coumaric acid–0.04 μg mg^−1^ Verbascoside-0.05 μg mg^−1^ Pinoresinol–0.32 μg mg^−1^ Apigenin-7-glucoside–0.13 μg mg^−1^ Rutin–0.05 μg mg^−1^ Hydroxytyrosol–0.06 μg mg^−1^	ND	CGA was the predominant polyphenol present in the extract	Simple and less costly process Energy can be saved due to extraction at room temperature Maceration avoids the costly and complex purification from the solvents To remove the solvent, rotary evaporation is needed Since ethanol is used as an extracting solvent, this method is not considered as a GEM Recovery of the solvents should be focused through condensing	[6,50]
Artichoke external bracts								
Diluted extracts (1:5 *w*/*v*) treated with enzyme-assisted extraction (EAE)-Pectinex Ultra SP-L (65 °C, pH 5.5), 0.45 (*v*/*w*) for 24 h	85.85 GAE mgg^−1^	ND	FRAP–130.47 µmol TEg^−1^ ABTS–83.58 µmol TEg^−1^ DPPH–129.20 µmol TEg^−1^	ND	86.97% extract	Treatment with different enzymes influenced the antioxidant capacity and the TPC of all the extracts. Extracts maintained a high yield following enzyme application, suggesting strong potential for large-scale implementation of this technology	EAE is a sustainable effective extraction method Moderately complex and costly process due to enzymes Energy can be saved due to extraction at reduced temperature EAE is a GEM and eco-friendly Extraction of high yield of bioactive compounds and beneficial for heat-labile compounds Prolonged treatment time and purification from the enzyme are drawbacks	[51]
Diluted extracts (1:5 *w*/*v*) treated with EAE-Viscozyme L (45 °C, pH 4.5), 0.45 (*v*/*w*) for 24 h	127.91 GAEmgg^−1^	ND	FRAP–182.12 µmol TEg^−1^ ABTS–126.10 µmol TEg^−1^ DPPH–217.02 µmol TEg^−1^		81.55% extract			
Diluted extracts (1:5 *w*/*v*) treated with EAE-Celluclast 1.5 L (50 °C, pH 6), 0.45 (*v*/*w*) for 24 h	109.2 GAEmgg^−1^	ND	FRAP–130.61 µmol TEg^−1^ ABTS–85.86 µmol TEg^−1^ DPPH–174.96 µmol TEg^−1^		80.24% extract			
Six different NADES mixtures of HBD and HBA (Choline chloride or betaine served as HBA, while sucrose, lactic acid, oxalic acid, glycerol, and citric acid act as HBD) were heated at 80 °C for 60–90 min. UAE–Treating in an ultrasonic bath at 60 °C for 30 min. Choline chloride: lactic acid–1:2	12.96 GAE kg^−1^ d.w.	ND	CUPRAC-60.68 g TE kg^−1^ d.w.)	CGA-3224 mgkg^−1^ d.w. Ferulic acid-2466 mg kg^−1^ d.w. Quercetin-499.6 mg kg^−1^ d.w. Gallic acid-154.8 mg kg^−1^ d.w. Rutin-142.1 mgkg^−1^ d.w. Syringic acid-51.0 mg kg^−1^ d.w.	ND	The effectiveness of obtaining higher TPC -ChCl-Suc > Bet-Ca > MeOH > Bet-La > ChCl-Ox > Bet-Glyc Although the phenolic profiles of the extracts were similar, the concentration of each phenolic compound varied according to the type of NADESs used NADESs composed of organic acids exhibit higher polarity than sugar- or polyol-based NADESs and water. This physicochemical property is associated with hydrogen bond formation and an enhanced ability to dissolve hydrophilic phytochemicals Identifying the best HBD and HBA combinations and maintaining the viscosity of the solvent mixture are important	Simple and less costly process Less energy consumption and environmentally friendly Since DESs have low volatility, it is difficult to remove the solvent from the extracts obtained.Therefore, the yield for downstream processes may be limited. To remove the solvents, phenolics should be adsorbed by resins, followed by the elution with an organic solvent	[52]
Artichoke leaves and stems								
Sequential extraction EAE using pectinase enzyme (Solid-to-liquid (S/L) ratio of 1:3, 3 h at 50 °C), followed by ethanolic extraction (60:40 ethanol to water ratio and a 1:10 S/L ratio) These steps were followed in the next extractions.	44.82 µmol GAE g^−1^ d.w. 301.40 µmol GAE g^−1^ dry extract (d.e.)	ND	ND	ND	14.87%	The significant performance of EUAE and its purified phenolic portion indicates that treatment combination either improves the recovery of phenolic compounds or enhances the extraction of components with strong antioxidant potential, which may be more heat labile and prone to degradation when using EMAE or EUMAE.	EAE is a sustainable and effective extraction method Moderately complex and costly process due to enzymes Energy can be saved due to extraction at reduced temperature EAE, MAE and UAE are GEMs and eco-friendly; however, since ethanol is used as an extracting solvent, the weight of being GEMs is reduced Suitable for extracting high yields of bioactive compounds and heat-labile compounds Prolonged treatment time and purification from the enzyme are drawbacks EAE followed by either MAE or UAE is more efficient in extracting phenolics Recovery of the solvents should be focused through condensing	[2]
EAE followed by MAE (EMAE) (isolated solid phase treated with 60% ethanol and a 1:10 S/L ratio, 5000 W for 7 min reaching a temperature of 60 °C)	180.09 µmol GAE g^−1^ d.w. 814.93 µmol GAE g^−1^ d.e After the purification -2643.21 µmol GAEg^−1^ d.e.	ND	ABTS–91.29 µmol TE g^−1^ d.e. DPPH–75.68 µmol TE g^−1^ d.e. FRAP–178.67 µmol TE g^−1^ d.e.	CGA isomers-53.41 mg g^−1^ d.e. Coumaric acid isomers–8.02 mgg^−1^ d.e. Caffeic acid–25.26 mg g^−1^ d.e. Feruloylquinic acid–4.56 mg g^−1^ d.e. Cynarin–10.79 mg g^−1^ d.e. Apigenin glucoside–50.98 mg g^−1^ d.e. Luteolin rutinoside–60.68 mg g^−1^ d.e. Hesperidin–36.69 mg g^−1^ d.e.	22.13% After the resin purification, the phenolic purity was 44.93%			
EAE followed by UAE (EUAE) (1200 W, 1 h at 30 °C)	210.76 µmol GAE g^−1^ d.w. 985.33 µmol GAE g^−1^ d.e. After the purification–2983.65 µmol GAEg^−1^ d.e.	ND	ABTS-80.46 µmol TE g^−1^ d.e. DPPH-87.03 µmol TE g^−1^ d.e. FRAP-184.99 µmol TE g^−1^ d.e.	CGA isomers-60.73 mg g^−1^ d.e. Coumaric acid isomers-7.58 mg g^−1^ d.e. Caffeic acid-34.29 mg g^−1^ d.e. Feruloylquinic acid-22.84 mgg^−1^ d.e. Cynarin-16.33 mg g^−1^ d.e. Apigenin glucoside-21.56 mg g^−1^ d.e. Luteolin rutinoside–103.27 mg g^−1^ d.e. Hesperidin-18.16 mg g^−1^ d.e.	21.38% After the resin purification the phenolic purity was 50.71%			
EAE followed by MAE (5000 W until 60 °C, 7 min) and UAE (1200 W, 35 min) (EUMAE)	211.35 µmol GAE g^−1^ d.w. 855.14 µmol GAE g^−1^ d.e. After purifying-2493.87 µmol GAEg^−1^ d.e.	ND	ABTS-63.81 µmol TE g^−1^ d.e DPPH-45.93 µmol TE g^−1^ d.e FRAP-186.87 µmol TE g^−1^ d.e	CGA isomers-47.07 mg g^−1^ d.e. Coumaric acid isomers-6.83 mg g^−1^ d.e. Caffeic acid-26.61 mg g^−1^ d.e. Feruloylquinic acid-12.04 mgg^−1^ d.e. Cynarin (1,3-dicaffeoylquinic acid)–1.78 mgg^−1^ d.e. Apigenin glucoside-21.59 mg g^−1^ d.e. Luteolin rutinoside-43.28 mg g^−1^ d.e. Hesperidin-6.92 mg g^−1^ d.e.	24.68% After the resin purification, phenolic purity was 42.39%			
External bracts, leaves, and stem								
UAE (MtOH: water (60:40, *v*/*v*) treating in an ultrasound bath for 30 min	29 mM GAEg^−1^ d.w.	ND	ABTS->25 mM TE g^−1^ d.w. DPPH->7mM TEg^−1^ d.w. FRAP->38 mM TE g^−1^ d.w.	Hydroxycinnamic Acids CGA–1006 µg g^−1^ d.w. Cynarin-12 µg g^−1^ d.w. Flavonoids Luteolin–4.5 µg g^−1^ d.w. Luteolin-7-O-glucoside-469 µg g^−1^ d.w. Luteolin-7-O-rutinoside-1034 µg g^−1^ d.w. Apigenin–2.49 µg g^−1^ d.w. Apigenin-7-O-glucoside–7.2 µg g^−1^ d.w. Apigenin-7-O-rutinoside–20.2 µg g^−1^ d.w. Naringenin-7-O-glucoside–2.9 µg g^−1^ d.w.	Hydroxycinnamic acids–31.2 µgg^−1^ Flavonoids–13.0 µgg^−1^	Luteolin derivatives are considerably more abundant in artichoke waste than in the edible portions, where apigenin derivatives—particularly apigenin-7-O-glucuronide—appear to be the predominant flavonoids. UAE increased the TPC only when 60% methanol was used as the extraction solvent.	Simple and less costly process Energy can be saved due to extraction at reduced temperature Mild treatment of ultrasounds does not produce any significant changes in the properties and functionality of most of the bioactive compounds, which makes it ideal for the extraction of sensitive phenolics UAE is a GEM; however, since methanol is a non-green and toxic solvent, it reduces its weight as a GEM Recovery of the solvents should be focused through condensing	[14,51]

ND—No data, TEAC-Total antioxidant capacity, TPC—Total polyphenol content, TFC—Total flavonoid content, GAE—Gallic acid equivalent, QE—Quercetin equivalent, TE—Trollox equivalent, CE—Catechin equivalent, dp—Dry plant, dw—Dry weight, de—Dry extract.

In solvent-based extraction, the concentration of the solvent plays a major role in both yield and bioactivity of the extracted polyphenols. Pure organic solvents including ethanol lower the extraction efficiency of phenolic compounds because of their hydrophilic nature, whereas ethanol enhances the extraction of cynaropicrin, which is a weakly polar compound extracted from artichoke leaves [47]. This highlights that selectivity is an important factor in choosing a solvent.

It is important to identify the best GEM for extracting targeted phenolic compounds. Considering the information presented in Table 1, NADESs (combined with ultrasonication) can be recommended as the most appropriate method to extract heat-labile hydrophilic compounds due to their ability to preserve the bioactivity while producing high yields (through combination with UAE), at reduced temperatures. Food-grade solvents are economically feasible, regenerative, highly recoverable, and mostly free from toxins.

Meanwhile, economically valuable hydrophobic compounds can be extracted by SCO_2_E as it will recover compounds with high purity, free from extracting solvents, and requiring no additional recovery steps. The process of SCO_2_E is comparatively highly expensive to establish; consequently, its applications are mainly limited to the extraction of high-value compounds.

EAE combined with UAE can be ideal to extract multiple bioactive compounds (fibres, polysaccharides, etc.) together with a single treatment, and the process conditions should be properly monitored for a maximum yield with preserved bioactivity. However, the recovery of the targeted compounds should be done under carefully monitored procedures.

### 3.1. Deep Eutectic Solvents (DESs) Assisted Extraction

DESs are an emerging class of solvents that have gained attention due to their unique properties and potential applications across various industries, particularly in extracting bioactive compounds from plant-based foods, with the increasingly attracted attention in recent years [53,54]. DESs are formed by combining a hydrogen bond acceptor (HBA) with a hydrogen bond donor (HBD) [45]. DESs interact through hydrogen bonding to create a eutectic mixture, the melting point of which is significantly lower than that of its individual constituents [55]. The preparation of these solvents is simple and straightforward, typically involving food-grade components such as organic acids (e.g., citric acid, lactic acid), glycerol, choline chloride, and salts of organic acids (e.g., sodium citrate) [28]. DESs are notable for their non-toxic nature, very low vapour pressure, adjustable composition, recyclability, and compatibility with food, pharmaceutical, and cosmetic applications [45]. Although DESs are comparable (in terms of physicochemical properties) to conventional ionic liquids such as imidazolium-based liquids, DESs are much more affordable and environmentally sustainable [55,56]. These distinctive properties render DESs highly suitable for establishing sustainable extraction processes from various plant-based food wastes.

When DESs solely comprise naturally derived substances, they are identified as NADESs. NADESs have emerged as promising green extraction media due to both environmental benefits and functional effectiveness. They are safe, non-toxic, biodegradable, and compatible with food and pharmaceutical uses. These solvents are easy and inexpensive to produce, and their key physicochemical characteristics, such as polarity, viscosity, conductivity, and melting point, can be adjusted by modifying the solvent combination or water content [57]. Since the solvents used for NADESs are classified as food-grade ingredients, it is not necessary to remove them from extracts formulated for human consumption [28]. Due to their extensive hydrogen-bonding networks, NADESs facilitate the solubility and stability of bioactive compounds via making bonds with extractants, even with limited water solubility. They are also suitable for many analytical methods, and their low volatility and potential for reuse (after isolating the bioactives) reduce the environmental footprint. Altogether, these properties make NADESs highly effective and sustainable solvents for natural product extraction [57,58]. The viscosity of NADESs is regarded as their most critical physicochemical property and represents one of the major challenges in employing eutectic solvents for extraction processes [59]. The effectiveness of the DES system is influenced by the proportion of water, as the addition of water can alter its physicochemical characteristics, such as polarity and viscosity, which play a crucial role in determining extractability and overall extraction performance [45,60]. Although increasing the DES-to-water ratio can improve the extraction yield, keep reducing the water content beyond a certain threshold becomes ineffective and challenging, as the solvent’s high viscosity makes the extract difficult to handle. In general, adding a specific amount of water (variable in different DES systems) to DES can be essential for properly regulating viscosity and polarity, while reducing the surface tension [59]. However, the water content must not exceed a certain limit (specific for the solvent combination), as excessive dilution can lead to DES disintegration and the loss of distinctive solvation properties, unavoidably influencing the extraction process and the resulting yield [61,62].

In an extraction process, time and temperature are interrelated factors that influence both the extraction yield and the composition of the extract. Although increasing the temperature can significantly enhance polyphenol extractability by reducing solvent viscosity, which promotes greater diffusion and solubilisation of polyphenols, in most cases, optimal temperatures generally do not exceed 80 °C [45,63]. Therefore, the inherent instability of heat-labile phenolics limits how much the temperature can be raised [63].

Studies indicate that phenolic compounds can be extracted more efficiently in their neutral form when the solvent pH is below the pKa of the phenolics. In Brahmi-Chendouh et al. [28], the TPC of artichoke waste extracted with NADESs (choline chloride and citric acid) followed the order, capitula > stems > leaves. Except for apigenin methylhexuronide, whose concentration was approximately 2.5-fold higher than the leaf sample, the capitula-based formulation exhibited the highest flavonoid content. Notably, flavonols were detected exclusively in artichoke heads. By contrast, the stem samples accounted for the most phenolic compounds, particularly caffeoyl- and dicaffeoyl-methylquinic acids [28].

TPC and antioxidant activity vary significantly, depending on the solvent type used. NADESs formulated with carboxylic acids such as lactic acid and citric acid exhibit higher polarity than those containing sugars or polyols, thereby favouring the extraction of polar polyphenols [52]. In contrast, NADESs made with choline chloride–oxalic acid and betaine–glycerol showed the lowest TPC values, which were even lower than conventional extraction. The lower antioxidant activity of extracts reported with non-organic acid representing mixtures might be linked with the variations in the phenolic composition or interference and contribution of non-phenolic compounds (vitamins, organic acids, etc.) [64]. However, apart from the hydrogen bond network, the other intrinsic factors behind the apparent changes in solubility and ability to extract remain unclear. Therefore, it is critical to discover the actual mechanism and select the ideal solvent combinations that develop strong interactions with the intended bioactive compounds which facilitate high solubility.

**Figure 2:**
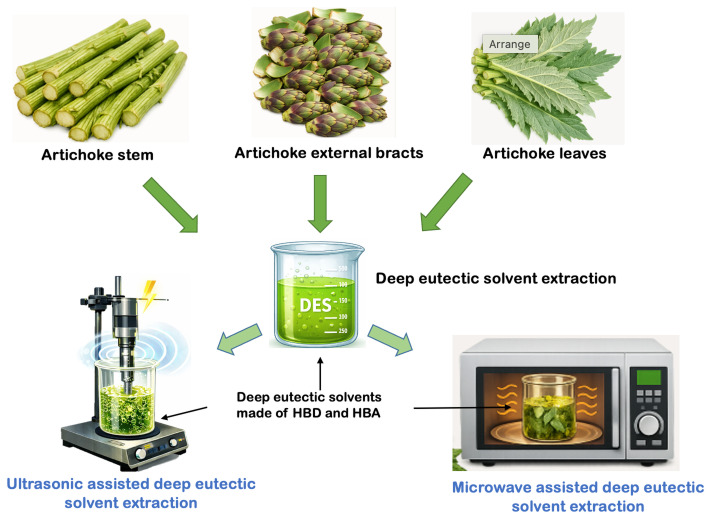
Ultrasonic-assisted DES extraction and microwave-assisted DES extraction of artichoke waste products.

Combining NADES-assisted extraction with other GEMs such as UAE and MAE is a promising avenue, as depicted in Figure 2. Owing to the low volatility of NADESs, their removal from the resulting extracts is challenging, which may restrict the recovery yield in downstream processing [52]. To overcome this limitation, phenolics can be selectively adsorbed onto resins and subsequently recovered through elution with an organic solvent. Different techniques, such as solid phase extraction, activated charcoal cleaning process, and antisolvent process, can be introduced to eliminate the NADESs and recover the extracted polyphenols [65]. Since most of the currently available recovery strategies are linked with either one or more non-green solvents, it is necessary to discover and develop green solutions by replacing conventional solvents with green solvents, while preserving the bioactivity of each compound.

As discussed above, the key advantage of NADESs over conventional volatile organic solvents, including methanol, ethanol, and acetone, is their natural origin and food-grade safety [66]. NADESs can be formulated from components designated as Generally Recognized as Safe (GRAS) by the FDA. This property makes them particularly suitable for food-related applications, as they substantially reduce toxicological concerns and minimise the risk of harmful residual contaminants to both human health and the environment [67]. However, some studies indicate that certain NADESs may exhibit toxicity (betaine:glycerol system) in rats [68]. Therefore, further studies are needed to fully understand the safety of each NADES mixture before introducing it into foods.

Since NADESs are produced with food-grade chemicals, unlike conventional solvents, NADESs are generally not removed from the final extract in most applications. Since a lack of dedicated regulatory guidelines from authorities such as the European Food Safety Authority (EFSA) and the U.S. Food and Drug Administration (FDA) has not yet been issued, the safety of extractants in food without isolating from the solvents remains questionable. Since in vivo and in vitro toxicological and biodegradability data remain insufficient (especially in the case of complex ternary or quaternary NADES systems), regulations should be formed based on more extensive studies.

### 3.2. Ultrasound Assisted Extraction (UAE)

UAE may be performed either by applying a probe directly to the sample or by using a water bath system in which ultrasonic energy is transferred indirectly through the container walls [32]. The operation of ultrasonic bath and probe treatments is based on the use of a piezoelectric transducer (applying an electric field to a material to vibrate) [69]. The probe technique can be operated in two modes: continuous and pulsed. In pulsed operation, the ultrasonic sonicator functions intermittently throughout the extraction process, with the total cycle time defined as the sum of the pulse on-time and interval time. The duty cycle is expressed as the proportion of the pulse on-time relative to the total cycle time [32]. UAE using a sonotrode (probe) is an innovative technique that shows strong potential for the recovery of bioactive compounds, as it allows higher extraction yields, shorter processing times, reduced solvent and energy consumption, while improving the extract quality [10,32,69]. The efficiency of the UAE in extraction is modulated by multiple parameters, notably extraction time, ultrasonic power, and solvent concentration. CGA extraction in 80% methanol using UAE with a probe is found to be more effective than extraction in an ultrasonic bath [70]. These results are consistent with those reported by Turksever et al. [32] for the TPC of artichoke leaves. This may be attributed to differences in extraction methods, as UAE with water bath operates at a single frequency (typically 20 or 40 kHz), whereas probe-based ultrasound delivers significantly higher ultrasonic power, up to 100 times greater, combined with direct exposure of sonication with the sample.

In Jimenez-Moreno et al. [14], UAE could enhance the extractability of phenolic compounds irrespective of the solvent, while methanol was more effective than water in extracting hydroxycinnamic acids and flavonoids from artichoke waste (stem, leaves and external bracts). Depending on the solvent used, its concentration and the presence or absence of ultrasonic treatment, the concentration of each extracted phenolic compound tends to differ. UAE can be an ideal method to extract 5-O-caffeoylquinic acid (CGA) in high amounts, as CGA was the main compound found in all samples with high levels. However, 1,3-O-dicaffeoylquinic acid (cynarin) (a compound supporting bile secretion and cholesterol metabolism) was present only in small amounts in 60% methanol extracts and absent in water extracts [71]. Masala et al. [47] also found the highest TPC from artichoke waste in UAE (3.02 mg g^−1^ dp), where 96% ethanol was used as the solvent, whereas the lowest TPC was detected in extracts obtained with 100% water (0.62 mg g^−1^ dp). These studies highlight that although being a green solvent, 100% water is not the best solvent media to extract phenolics from artichoke waste, as it contains semi-polar and non-polar compounds along with the polar substances.

The effective diffusion coefficient of UAE is influenced by temperature, while both the external mass transfer coefficient and the equilibrium extraction yield are affected by temperature as well as ultrasound power density [10]. Although probe-based ultrasonication has a significant effect over ultrasonic bath, the processing mode (either continuous or pulsed) does not have a significant effect on TPC or TFC when treating under constant temperature at 50 °C [32]. This outcome was likely due to the maintenance of a constant temperature in both modes, with similar results observed after extraction times of both 15 and 30 min.

Since bioactive compounds extraction is limited by thermal sensitivity, with temperatures above 75 °C causing substantial degradation and conventional mechanical agitation is often ineffective in releasing phenolics that are ester-bound or entrapped within cell wall proteins and polysaccharides [10,72], UAE can serve as a viable alternative to conventional extraction techniques by alleviating these inherent drawbacks. In the mathematical model presented by Reche et al. [10] with artichoke waste, the main resistance to molecular diffusion arises from cell walls and membranes separating the intercellular space from the liquid phase. Accordingly, their broken-and-intact cell model describes extraction kinetics as governed by both convective and diffusive mass transfer mechanisms. The relative contribution of these mechanisms depends on extraction conditions (agitation or ultrasound assistance and temperature) and may vary over time as ultrasound-induced structural changes modify the plant matrix.

Although the ultrasonic probe system is highly efficient, it is restricted to processing small sample volumes. In contrast, ultrasonic baths are simple to operate and cost-effective; however, they face challenges in terms of scalability and reproducibility [69]. Despite various advantages of the ultrasonic probe, its application remains limited by the small volume it can handle. For industrial-scale UAE, the nature of the product is a critical consideration. Larger processing capacities can be achieved either through continuous systems with limited reactor volumes or by employing ultrasonic baths with expanded radiating surfaces [69]. Integrated GEMs (e.g., NADES + UAE) can be more sustainable and effective in recovering high yields of bioactive compounds while preserving the inherent bioactivity of the extract. Therefore, further studies should be focused on novel approaches for the possible integration of extraction techniques, while identifying potential scaling-up opportunities.

### 3.3. Microwave Assisted Extraction (MAE)

MAE is associated with shortened processing times, reduced solvent and energy consumption, and the production of high-quality extracts with improved extraction yields [32]. Microwave heating with high-frequency electromagnetic microwaves (300–300,000 MHz) occurs through the direct interaction of microwaves with polar molecules such as water and certain organic components of plant matrices via ionic conduction and dipole rotation mechanisms [32,55]. In microwave heating, mass and heat transfer occur simultaneously in the same direction, creating a synergistic effect that enhances the extraction rate while improving the overall yield [55,73]. MAE shortens extraction time and preserves heat-sensitive compounds, making it particularly suitable for isolating polyphenols [46]. This method employs electromagnetic radiation that acts directly on molecules via dipole rotation, ionic conduction, or both. As the microwave frequency increases, polar molecules like water attempt to align with the oscillating electric field, resulting in rapid molecular rotation, leading to rapid internal heating and enhanced mass transfer driven by the pressure generated from water vapour inside the cells [74]. This internal pressure weakens and ultimately ruptures the cell walls, facilitating the release of intracellular compounds into the extraction solvent [46,75]. Since different compounds absorb electromagnetic energy to varying extents, MAE enables the selective extraction of bioactive components from complex food matrices. The absorption of microwave energy and the corresponding rise in temperature within the sample depend on the dielectric loss factor and the square of the electric field strength. An increase in microwave power amplifies the electric field strength, leading to a substantial rise in the sample’s temperature [46]. Higher extraction temperatures lead to greater intracellular pressure, lower diffusion resistance, and enhanced solubility of anthocyanins. The elevated temperatures decrease solvent viscosity, facilitating more efficient diffusion of anthocyanins into the extraction medium [46].

In Turksever et al. [32], MAE showed the highest efficiency (with respect to TPC in artichoke leaves) over other extraction techniques (UAE and maceration). This enhanced performance can be attributed to the rapid interaction between microwave energy and solvent molecules, which accelerates cell wall disruption and facilitates the release of bioactive compounds.

### 3.4. Enzyme Assisted Extraction (EAE)

Enzymatic hydrolysis is considered a highly promising technique owing to its superior product yield and low energy demand [76]. Although it is generally more expensive than physical methods due to enzymes and their purification, it is regarded as an environmentally friendly approach, as it avoids the use of organic solvents and chemical reagents [51]. In Thang et al. [77], EAE using cholesterol esterase, cellulase, and pectinase significantly enhanced the yield of cynarine and CGA from artichoke leaves compared to conventional (ethanolic extraction) and UAE, while pectinase provided the highest yield. Antioxidant activity, along with antimicrobial effects against *B. cereus*, *E. coli*, and *P. aeruginosa*, highlighted their significance for potential pharmaceutical applications.

In Ayuso et al. [51], the extraction of polyphenols through EAE was most efficient with Viscozyme L (enzyme cocktail mixture of arabanase, xylanase, β-glucanase, cellulase and hemicellulase from *Aspergillus aculeatus*) (50.87%), followed by Celluclast 1.5 L (cellulase from *Trichoderma reesei*) (28.80%). Results showed that the most effective enzyme to extract fibre may not be the best to extract polyphenols, highlighting the need to identify the ideal enzyme and the optimum treatment conditions, prior to incorporating into mass production. In contrast to other extraction methods, enzymatic extraction can be highly successful in extracting many different bioactive compounds at once, using less time, energy, and resources. Precise purification and recovery steps are needed to separate the bioactive compounds from the mixture, through controlling the process conditions or by advanced purification techniques.

Sequential extraction strategies employing a two-step approach have been more effective than conventional single-step methods, yielding higher bioactive compounds, including more biologically active polyphenols and flavonoids [78]. Gil-Martinez et al. [2] studied the effectiveness of enzymatic pre-treatment combined with MAE, UAE, and hybrid UAE and MAE at the pilot scale, for the recovery of antioxidant compounds from artichoke waste and reported higher amounts compared with any single treatment.

Sabater et al. [79] investigated the application of Celluclast 1.5 L to artichoke external bracts, leaves, and stems, showing a strong fibre-hydrolysing capacity and enhanced pectin extraction. Similarly, Fissore et al. [80] also observed that enzymatic treatment of artichoke by-products increased inulin levels, the principal component of artichoke soluble dietary fibre. During pectinase-assisted extraction, the breakdown of pectin within the primary cell wall matrix and the middle lamella is essential for disrupting its water-binding capacity, thereby enhancing the hydration properties of the samples. This effect is attributed to the exposure of hydroxyl groups, which facilitate water interactions through hydrogen bonding. Since the same mechanism works for phenolic compound extraction, it is important to analyse the suitability of various fibre-hydrolysing enzymes and optimum process conditions to introduce sequential extraction.

Enzymes can selectively target and break specific bonds within plant materials, enabling a more precise and efficient extraction of desired bioactive compounds [81]. Enzymes hydrolyze the glycosidic bonds in the main chains of polygalacturonic acid within cell walls [82]. Celluclast 1.5 L catalyses the hydrolysis of β-1,4-glycosidic bonds in cellulose and hemicellulose, exhibiting cellulolytic, xylanolytic, and mannanolytic activities [51]. The process conditions and separating methods can ideally be applied for retrieving phenolic compounds as the initial stage, inulin and pectin as the next stage and fibres as the third stage. Since sequential extraction facilitates a sustainable approach for utilising artichoke waste through EAE, heat or chemically sensitive compounds present in plant masses can be safely extracted and isolated for high-value product development.

Although EAE is considered a non-thermal extraction method, inactivation of the enzyme at higher temperature (e.g., 80 °C) [77] for a few minutes shows degradation of heat-labile phenolic compounds, highlighting one of the potential drawbacks. Therefore, enzymes that can be inactivated under low heat and temperature, or other process conditions, should be introduced for optimal bioactivity preservation. EAE is considered less cost-effective due to several factors, such as the high cost of the enzymes, the need to have specific treatment chambers, and professional knowledge to handle the process, which appear as limitations and drawbacks when applying on a commercial scale. Immobilisation of enzymes can help overcome several associated limitations, including enzyme inactivation caused by molecular aggregation, poor stability, lack of reusability, difficulties in recovery, and the high cost of enzymes [83,84]. Additionally, immobilisation can enable continuous operation, improving process control, supporting cost-effective production, and minimising enzyme inhibition [83,85]. Although enzyme immobilisation requires several additional steps, when extracting and isolating valuable compounds and applying them in food product development, it can improve the economic feasibility of EAE at an industrial scale. In future research, a life cycle assessment (LCA) can be performed to generate the validity and feasibility of EAE.

### 3.5. Supercritical Carbon Dioxide Extraction (SCO_2_E)

A substance can enter the supercritical fluid (SCF) state when it is exposed to conditions above its critical temperature and critical pressure. Under these conditions, the physicochemical properties of the fluid become intermediate between those of gases and liquids. This distinct state exhibits properties such as gas-like permeability and liquid-like solubility [86]. SCO_2_E is a highly versatile technique, as its efficiency can be tuned by adjusting the temperature or pressure, which in turn modifies the CO_2_ density and solubility of target compounds. This method is particularly effective for isolating high-value compounds from non-polar to moderately polar molecules, such as essential oils.

Masala et al. [47] studied various GEMs, including UAE, DESs, SCO_2_E and subcritical water extraction (SWE), and out of these, the extract from SCO_2_E contained the highest levels of the sesquiterpene lactones cynaropicrin (48.33 mg g^−1^ dp) and cynaroscoloside A/B (8.22 mg g^−1^ dp). SCO_2_E has a limited ability to extract polar compounds [87]. Notably, the TPC of samples in Masala et al. [47] was relatively low at 1.53 mg g^−1^ dp, with only luteolin 7-O-rutinoside and luteolin 7-O-glucoside being identified and quantified. The total hydroxycinnamic acid (THC) content was low (0.08 mg g^−1^ dp), and no hydroxybenzoic acids were detected in the extract. This is a significant limitation in applying SCO_2_E as a technique to extract bioactive compounds, as a considerable amount of artichoke phenolics are relatively polar.

Polar modifiers or cosolvents such as ethanol, acetonitrile, methanol, and n-hexane can be introduced to SCO_2_E to improve the solubility and extraction of polar bioactives [47,88]. In this method, choosing a suitable modifier, without compromising the efficiency of CO_2_ in extraction, is essential for optimising the extraction parameters. Their findings demonstrated that the increase in extraction efficiency up to 33 MPa was reasonably attributed to higher CO_2_ density and the solubility of the solvent; however, beyond that pressure level, dense CO_2_ has damaged the interactions between the solute and the solvent, leading to increased mass transfer resistance within the plant matrix. Similarly, although increasing the modifier volume enhanced the extraction of polar compounds, excessive modifiers more likely altered the polar conditions, lowering the extraction efficiency. Therefore, it is important to monitor the process conditions to optimise the extraction without compromising both bioactivity and yield.

SCO_2_E can also be combined with other GEMs to recover polar, semi-polar and non-polar phenolic compounds. First, the polar and semi-polar compounds can be extracted using appropriate green solvents and then the dry remainder can be treated with SCO_2_E to extract and recover the nonpolar compounds. Future studies are suggested to identify the feasibility and potential to combine SCO_2_E with other GEMs for optimum recovery of bioactives. A thorough understanding of the thermodynamic and kinetic principles underlying SCO_2_E is essential for achieving high selectivity in the extraction process while minimising the co-extraction of non-target compounds [87]. Requiring expert knowledge and expensive instruments, and the limited solubility of the polar compound in CO_2_ are drawbacks in implementing SCO_2_E as an effective technique for extracting bioactive compounds from artichoke waste.

### 3.6. Pulsed Electric Field Assisted Extraction (PEFE)

Pulsed electric field (PEF) treatment has emerged as a promising and efficient alternative to conventional cell-disintegration methods such as sonication, grinding, milling and osmotic shock [74,89]. PEF application can reduce the reliance on energy-intensive mechanical size reduction, allowing the use of larger biomass particles while maintaining compound integrity and improving downstream processing efficiency [90]. During PEF processing, plant tissues are subjected to microsecond pulses of moderate electric field strength (0.5–10 kV cm^−1^), inducing electroporation of cell membranes, enhancing the release of intracellular constituents while preserving the particle size of the raw material. In Carullo et al. [90], membrane permeabilization increased with higher field strength and energy input, showing greater effectiveness above 0.5 kV cm^−1^ and in larger particles, before reaching saturation at around 5 kV cm^−1^ (5 kJ kg^−1^). Meanwhile, Carpentieri et al. [30] recorded the optimum phenolic extraction at 3 kV cm^−1^ with the same energy input. Such treatment has demonstrated strong potential for boosting the selective extraction of valuable intracellular compounds, including polyphenols from diverse food processing wastes and by-products, while simultaneously lowering energy usage, solvent requirements, and overall processing time [89,90]. A larger surface area exposed to electrical pulses results in more extensive cell disruption, highlighting the improved effectiveness of applying PEF after the size reduction [74].

The extent of cell disruption is influenced by treatment intensity and sample size, indicating that PEF conditions can be optimised to enhance the extraction efficiency of phenolic compounds [90]. In Carullo et al. [90], increasing the intensity of comminution enhanced the diffusion rates by promoting partial cell disruption, which resulted in higher TPC and improved antioxidant activity. The application of PEF further facilitates the release of phenolic compounds, leading to significantly greater yields. However, the effectiveness of PEF diminishes with increased cutting intensity, as smaller particle sizes contain fewer intact cells available for electroporation.

Although PEFE can be a very effective GEM in extracting bioactive compounds, it needs a moderately expensive setup, highlighting a potential drawback in applications. Although the extraction is cost-effective, only a few studies have been conducted for extracting bioactives from artichoke and its by-products. Therefore, future research is recommended to identify the best process conditions to optimise the yield while preserving the bioactivity and targeted compounds.

### 3.7. High Pressure Assisted Extraction (HPAE)

HPAE consists of three main processing stages: pressure boost, maintaining, and relief stages [91]. During the pressure boost stage (first stage), the pressure rapidly increases from atmospheric to the desired processing level within a short period. This sudden rise in pressure disrupts cellular structures, thereby enhancing the mass transfer of phenolic compounds. The efficiency of mass transfer is proportional to the applied pressure and the resistance to diffusion. In the maintaining stage (second stage), the solvent penetrates the cells more effectively because of the structural damage caused during the initial stage, promoting further extraction of phenolic compounds. Finally, in the pressure release stage (third stage), the pressure is reduced from the operating level back to atmospheric pressure. This decompression induces significant alterations in hydrogen bonds, ionic bonds, and hydrophobic forces that maintain the polymeric structures of the cells. Consequently, the cells expand and develop porous, ruptured, or loosened structures, facilitating improved diffusion and recovery of bioactive compounds [91], allowing the extraction solvent to penetrate the cellular structure and interact with the bioactive constituents. As the pressure increases, a greater amount of solvent diffuses into the cells, resulting in improved recovery of bioactive compounds [91].

Extraction time and the solid-to-solvent ratio of the extractants are also crucial in HPAE of phenolic compounds. Okur et al. [91] indicated that the TPC levels of olive leaves rose as the extraction time increased from 5 to 15 min when other conditions were unchanged. Likewise, Briones-Labarca et al. [92] reported that phenolic content in discarded blueberries increased by approximately 21% when the extraction time was extended from 5 to 15 min at 500 MPa. Lowering the solid/solvent ratio enhanced the extraction of phenolic compounds from olive leaves when extracted using HPAE [91]. Jun [93] also reported that the extraction yield for phenolic compounds from green tea using HPAE increased from 17% to 30% when the liquid-to-solid ratio was elevated from 10 to 20 mL g^−1^ at 500 MPa. These studies demonstrate that reducing the solid-to-solvent ratio increases the interaction between bioactive compounds and the solvent, resulting in higher extraction efficiency due to enhanced leaching of the polyphenols.

Although studies have been conducted for extracting polyphenols from various plant waste products using HPAE, no research was found regarding HPAE in extracting artichoke waste. This highlights the importance and opportunities for future research focusing on the effectiveness of HPAE in the valorisation of artichoke waste.

### 3.8. Subcritical Water Extraction (SWE)

SWE takes advantage of the distinctive properties of water under subcritical conditions, such as a reduced dielectric constant, enhanced diffusivity, and increased ionisation constant, resulting in significantly higher phenolic extraction yields compared with conventional methods such as solvent extraction, maceration, etc. [94]. Masala et al. [47] studied the SWE at high temperature (125–250 °C) and found that when artichoke leaves were extracted with 100% water under elevated temperatures, flavonoids were prone to thermal degradation, rendering them undetectable. Orbenes et al. [95] also employed SWE within a temperature range of 140–240 °C and demonstrated that the highest TPC values, 2.9 and 3.8 g GAE 100 mg^−1^, were obtained from artichoke leaves at 220 °C. Although SWE generally utilises higher temperatures (>120 °C at high-pressure conditions), producing high yield, there may be a significant degradation of heat-labile bioactive compounds, including polyphenols. Therefore, SWE can only be recommended for heat and high-pressure-resistant targeted compounds. Since high temperature and pressure are applied, the safety of the extraction premises should be secured, avoiding any potential damage.

When adopting GEM into industrial-scale production, it is required to address a number of practical issues, including lowering the costs, as GRAS approved reagents remain comparatively costly, establishing standardised synthesis methods and consistent quality control standards, and conducting comprehensive risk assessments and food safety evaluations [66]. Therefore, adequate evaluations are needed before introducing a suitable GEM to any industrial production.

### 3.9. Life Cycle Assessment (LCA) of GEMs Against Conventional Extraction Methods

The environmental impact of the extraction methods is commonly assessed using LCA indicators, including carbon footprint, water consumption, energy use, and waste production. A major benefit of GEMs is their reduced carbon footprint relative to conventional extraction techniques such as Soxhlet and solvent-based extraction, typically involving high operating temperatures with extensive energy utilisation, lengthy extraction durations, and the extensive use of fossil fuel–derived organic solvents, all of which significantly increase greenhouse gas emissions [96].

In comparison, GEMs such as UAE, MAE, SCO_2_E, and EAE are usually carried out under milder processing conditions and consume less energy, leading to lower carbon emissions [97]. LCA shows that replacing 24 h maceration with UAE could reduce energy use by ~70% and CO_2_ equivalent emissions by ~60%, mainly due to lower extraction time and solvent demand [98]. SCO_2_E also employs recyclable carbon dioxide as a solvent, which helps reduce environmental pollution and solvent disposal problems [47].

Water consumption is also an important LCA parameter used to evaluate sustainability. Conventional extraction processes often require substantial amounts of water for processing, condensing, equipment cleaning, and solvent recovery. This excessive usage can strain freshwater resources and produce contaminated wastewater that requires additional treatment [96]. GEMs address this issue by incorporating solvent-free or water-efficient technologies [1,47]. Methods such as HPAE and EAE can decrease the need for intensive washing and solvent purification, thereby minimising overall water usage and wastewater production [99].

Beyond their environmental advantages, GEMs also enhance resource utilisation and reduce waste generation [43]. Conventional extraction techniques often generate large quantities of hazardous waste because of necessary solvent evaporation and chemical disposal processes. In contrast, GEMs emphasise the use of biodegradable solvents, renewable feedstocks, and recyclable processing systems, thereby lowering environmental hazards and promoting sustainable operations [2]. Additionally, many GEMs provide improved extraction yields and reduced processing times, contributing to greater industrial efficiency and economic viability [96].

However, despite these benefits, the adoption of GEMs still faces several challenges. Many advanced GEMs involve substantial initial investment, require specialised instruments, and demand technical expertise for operation and maintenance. Furthermore, some GEMs have not yet achieved full scalability for large-scale industrial implementation. Although GEMs generally exhibit better environmental performance according to LCA studies, economic and operational considerations remain important factors influencing their broader industrial application [2,96].

## 4. Encapsulation of Phenolic Compounds

The effectiveness of phenolic compounds in food and pharmaceutical applications is strongly influenced not only by the extraction method used to recover them from plant materials, but also by the encapsulation technique applied to protect and deliver them. Efficient extraction methods are essential for obtaining high yields of bioactive phenolics while preserving their functional properties, whereas encapsulation technologies further enhance their stability, bioavailability, and controlled release.

Phenolic compounds are known to exhibit limited stability and low bioavailability in the gastrointestinal tract, and they are highly sensitive to environmental factors such as light, temperature, pH, oxygen, and enzymatic activity [100]. Consequently, encapsulation strategies have been widely investigated to protect these compounds, maintain their integrity, and modulate or improve the release characteristics [101,102]. Microencapsulation is widely used to protect functional food components and control the release of compounds such as polyunsaturated fatty acids (PUFAs), antioxidants, flavours, and vitamins, while enhancing their bioaccessibility [103,104,105].

Selecting an appropriate microencapsulation technique and wall material is essential to ensure efficient ingredient encapsulation, as well as to achieve the desired functionality and stability of the resulting capsules [103]. Factors such as the characteristics of the core material (e.g., molecular weight, electrical charge, solubility, melting point, volatility, and sensitivity to heat and light), the intended application, and the potential for scale-up help to determine the most appropriate encapsulation technique for each specific case [103]. Table 2 demonstrates several encapsulation techniques used for encapsulating polyphenols extracted from artichoke waste.

### 4.1. Coacervation

Coacervation offers several advantages in microencapsulation, including exceptionally high encapsulation efficiency (EE) (up to 99% in hazelnut protein isolate-sodium alginate/quercetin 4:1, pH = 3.5, zeta potential = 7.24) under operation at low or ambient temperatures, cost-effectiveness, and the absence of a need for specialised equipment or toxic solvents [52,103,109]. Complex coacervation employs proteins as the primary wall material, combined with polysaccharides, to encapsulate a wide range of compounds and molecules. This technique is especially well-suited for use in the growing functional-food industry due to the biocompatibility and affordability of its coating materials [103]. Complex coacervation also offers several benefits, including high EE, the need for only a small amount of wall material, and the suitability for controlled release applications. The process typically involves three main stages: emulsification, coacervation, and shell formation or hardening [104].

Although animal-based protein sources such as milk proteins can be used, the limitation of the coacervation process is the partial denaturation and conformational changes in proteins occurring during emulsion preparation (ultrasonication, heating, and high-pressure treatment). These alterations can adversely affect the formation of coacervates. Since the food industry is increasingly aiming to reduce the use of animal-derived products, and milk proteins are recognised as potent allergens, plant proteins can be the best replacement [110].

Plant proteins are considered environmentally friendly, inexpensive, readily available, and possess attractive functional properties. Soy protein (SP) exhibits desirable characteristics for encapsulation, including emulsifying ability, solubility, film-forming properties, and water-binding capacity. In addition, it has a high nutritional value, containing at least 90% protein and being nearly free of lipids and carbohydrates [111]. Owing to their amphipathic nature (with both hydrophilic and hydrophobic regions), these proteins can effectively diffuse and/or adsorb at the oil–water interface during emulsification. As a result, they function as efficient emulsifiers that promote the formation and stabilisation of oil-in-water emulsions [112]. Nevertheless, the hydrophilic–hydrophobic balance on the protein surface also influences protein solubility, which is an important factor in complex coacervation. Although SP naturally exhibits relatively low solubility, this limitation can be improved through the addition of polymers such as Xanthan gum.

Complex coacervation is based on the associative interactions between oppositely charged macromolecules [46]. This is influenced primarily by the pH and the isoelectric point of each biopolymer, along with other factors such as ionic strength, the protein-to-polysaccharide ratio, and temperature. Under neutral pH conditions, the polymers typically remain co-soluble [103]. The formation of protein–polysaccharide complex coacervates generally occurs within the pH range between the pKa of the polysaccharide’s functional groups and the isoelectric point (pI) of the protein [103,104]. The protein/polysaccharide ratio that exhibited the greatest turbidity and a neutral zeta potential can be identified as the optimal ratio. Unlike protein precipitation, which depends solely on strong interactions causing proteins to separate from water molecules, protein–polysaccharide coacervation requires achieving electrical neutrality [103]. Complex coacervation enables high EE, with the potential to load core materials above 50% [110]. The method is well-suited for low-temperature processing and offers controlled release capabilities. In this method, the pH-responsive shielding effect, along with thermal and oxidative protection, safeguards phenolics from adverse external conditions.

### 4.2. Spray-Drying

This technique enables the rapid transformation of liquid feed materials into dry microparticles under controlled conditions, helping to preserve the functional properties of bioactive compounds. Compared with other microencapsulation approaches such as freeze-drying or coacervation, spray-drying offers several advantages, including faster processing, lower operational costs, applicability to both heat-sensitive and heat-stable materials, flexibility in production scale, and the ability to precisely control particle size [111,112].

Spray-drying microencapsulation technology is based on the formation of a protective barrier (membrane or shell) surrounding the core material, which contains the active ingredient to be encapsulated [112]. Selecting appropriate wall material for spray-drying microencapsulation is a critical step, as it directly affects EE, mechanical integrity, and the shelf life of the final encapsulated product [113]. The suitability of wall material is primarily determined by its high solubility in aqueous systems, effective emulsifying capacity, film-forming ability, and cost-effectiveness [112]. The common wall materials used in the food and nutraceutical industry are maltodextrin, gum Arabic, chitosan and different protein isolates [114,115,116].

Iervese et al. [101] studied the ethanolic phenolic-rich extracts derived from artichoke bracts, stems, and leaves for their use in formulating oil-in-water emulsions, as well as for their encapsulation through spray-drying to enable application in a practical emulsified system. In these emulsions, the protein level required for emulsion stabilisation should be determined by evaluating the capacity of pea protein concentrate to stabilise oil–in–water emulsion. The incorporation of pea proteins into emulsions can promote flocculation, which may lead to rapid system destabilisation by enhancing gravitational separation, an outcome that is generally undesirable in emulsion systems [117]. In Iervese et al. [101], the addition of artichoke extracts demonstrated promising potential to modulate the flocculation index likely due to bioactive compounds in the extracts, particularly phenolics, which have a strong affinity for proteins, significantly decreasing flocculation while, in most cases, not markedly affecting the particle size. Spray drying encapsulation of the leaf extract using maltodextrins as the wall material influences the release behaviour of phenolic compounds in a commercial vegan mayonnaise under simulated gastrointestinal digestion. The encapsulated extract exhibited a protective effect in the gastric phase (control with 56.48% and encapsulated samples with 13.36% of release at pH = 2–3) and promoted a greater release during the intestinal phase, thereby potentially enhancing phenolic absorption and bioaccessibility. Maltodextrin’s ability to reduce bitterness in artichoke leaves (particularly cynaropicrin accounts for nearly 80% of the bitterness) can be an additional benefit when incorporating the encapsulated polyphenols (coated with maltodextrin) into food [118,119].

**Figure 3:**
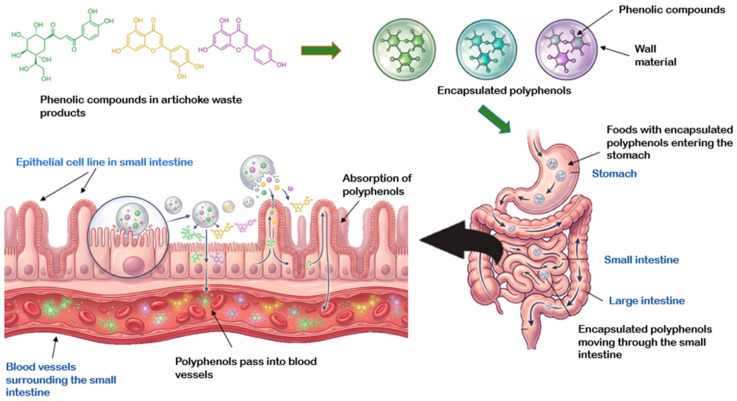
The mechanism of release and absorption of encapsulated polyphenols in the gut (created using ChatGPT 5.5 and PowerPoint).

For polyphenols to exert biological activity, they must first be bioaccessible within the gastrointestinal tract and then absorbed in the small intestine, enabling their entry into systemic circulation and subsequent distribution to target tissues and organs. Encapsulated polyphenols should be available at the absorbing spots in the intestine for their maximum bioaccessibility (Figure 3). The wall material should have a maximum resistance to gastric conditions to enable higher retention of polyphenols within the capsule and facilitate release at the epithelial cells in the small intestine. Application of wall material such as maltodextrin prior to spray-drying can be a positive initiative to preserve bioactive compounds until they reach the targeted area in the gastrointestinal tract.

### 4.3. Ionic Gelation

While EE is a primary criterion for establishing and optimising an encapsulation process, variations in the method can also lead to changes in the physical properties of the beads, including their size, appearance, and mechanical strength [102]. Alginate is a naturally derived, anionic, hydrophilic linear biopolymer that is widely used as an encapsulation matrix for protecting unstable bioactive compounds [120]. In the presence of divalent cations such as Ca^2+^, crosslinking takes place with alginate, resulting in the formation of a hydrogel that is widely used to immobilise bioentities, most commonly in bead form [102]. When an alginate solution droplet contacts a Ca^2+^ containing gelling bath, surface gelation is initiated, leading to the formation of a spherical structure as the system minimises surface tension [102]. Consequently, an initial Ca(II)–alginate network forms and reorganises at the periphery of the original droplet, while free Ca^2+^ ions diffuse through this gel layer toward the bead interior, progressively inducing gelation in a concentric way and resulting in a highly inhomogeneous microstructure [121]. In Sonego et al. [121], Ca(II)- and Ce(III)-alginate samples produced through cation exchange from homogeneous alginate hydrogels preserved both the multiple junction structural arrangement and the uniform microstructure of the original hydrogel, compared to conventional methods. This finding is significant because the approach enables the formation of homogeneous hydrogels, avoiding the structural inhomogeneity typically caused by cation concentration gradients and alginate chain migration during gelation. In Stoppel et al. [122], inhomogeneously cross-linked hydrogels (externally cross-linked with CaCl_2_ or BaCl_2_) had the effective diffusivity (D_eff_) increased by up to 50% for riboflavin and 83% for proteins, in contrast, protein transport in homogeneously cross-linked hydrogels (represented by BSA) was significantly influenced, with up to a 43% reduction in loading capacity and a 40% increase in D_eff_. It can be assumed that the smaller pore size on the outer surface of inhomogeneously cross-linked hydrogels, compared with homogeneously cross-linked hydrogels, accounts for 30% reduction in the overall release percentage [122]. These findings strongly indicate that the selection of the gelation method is crucial for targeting the encapsulating compound and its intended release.

Although it can be expected that prolonged gel consolidation time tends to decrease the EE of bioactive compounds by promoting their diffusion into the gelation bath, the study of Zazzali et al. [102] observed that gel consolidation time did not exert a significant effect. Meanwhile, Aguirre Calvo et al. [120] observed visible coloration due to pigment diffusion, with prolonged residence of the beads in the gelling bath. Conversely, higher alginate concentrations, particularly under extended consolidation times, resulted in larger and more compact rod-like microstructures [123], strengthening the interactions between the gel matrix and the encapsulated bioactive compounds. Interestingly, despite the relatively short consolidation times investigated in Zazzali et al. [102] with artichoke waste products, gels consolidated for longer durations (2–6 min) exhibited greater strength compared to the control, likely due to increased concentration of Ca^2+^ cations (2.50% (*w*/*v*)), which promotes a higher degree of chain crosslinking.

In both Jeong et al. [124] and Zazzali et al. [102], the alginate solution concentration was observed to be directly proportional to bead strength, likely due to the formation of a more compact and consolidated internal structure, as evidenced and elucidated by microstructural parameters. Moreover, a strong positive correlation between alginate concentration and roundness was observed in Zazzali et al. [102]. The ability to encapsulate CQA, CQA, and two dicaffeoylquinic acid isomers in all samples confirmed the successful preservation throughout the encapsulation processes. The strength and the thickness of the beads should be optimised, focusing on the intended use. The release of polyphenols at the targeted spot and the conditions in the gastrointestinal tract play a critical role when designing the beads for the food or nutraceutical industry. It is important to maintain the pH of the medium throughout the bead making for their strength and stability. Future research can also focus on the prospects of these approaches for achieving efficient encapsulation through appropriate synergism of several encapsulation methods.

### 4.4. Liposome Entrapment

Conventional nanoencapsulation materials present several limitations, including high cost, the requirement for elevated levels of surfactants or cosurfactants for stabilisation, susceptibility to environmental conditions such as temperature, pH, and ionic strength, and limited capacity to simultaneously protect both hydrophilic and hydrophobic phenolic compounds within the extracts [125]. One approach to enhance the effectiveness of compounds with low bioavailability while minimising the required dosage is their incorporation into designed delivery systems such as phospholipid vesicles, and when prepared using food-grade materials and processes, these systems are particularly suitable for oral administration [126]. Liposomes are primarily composed of phospholipid bilayers forming vesicular structures capable of encapsulating both hydrophilic and hydrophobic phases. Evidence suggests that incorporating phenolic compounds into liposomes enhances their therapeutic efficacy by improving cellular uptake and prolonging intracellular retention [127,128]. Liposomes exhibit a strong capacity to act as delivery vehicles for phenolic compounds, enabling efficient transport within the digestive tract while facilitating sustained release and enhanced functional efficacy by protecting encapsulated compounds from degradation caused by factors such as pH variations, light exposure, and digestive enzymes [127,129].

It was observed that formulations with lower EE exhibit greater release of phenolic compounds, likely due to effective encapsulation within vesicles delaying their release [130,131]. Furthermore, under more alkaline conditions, phenolics in aqueous extract more readily dissociate from the lipid matrix, facilitating their release [132].

In Castangia et al. [107] and Cassini et al. [132], the liposome behaviour closely corresponded to pH-induced changes in vesicle size. Conventional liposomes (liposomes only with artichoke extract and phospholipids) were more susceptible under acidic conditions, undergoing structural reorganisation or disruption that led to the release of their encapsulated contents in the stomach. In contrast, zein-based liposomes maintained structural integrity in acidic environments but gradually reorganised or disintegrated at neutral pH (7.0), resulting in a slower release profile in the intestine, facilitating more bioaccessible phenolic compounds. Zein, a polypeptide rich in non-polar amino acids (>50%), exhibits predominantly lipophilic characteristics and provides stability against degradation under acidic conditions. These liposome systems in Castangia et al. [107] had inhibitory effects on α-amylase and α-glucosidase activities, contributing to the protection of intestinal cells against oxidative damage.

Although liposomes are efficient in delivering phenolic compounds until they reach the intestine, Zein-incorporated liposomes impart the optimum protection, being resistant to conditions in the stomach. Zein-based liposomes can be successfully freeze-dried for long-term storage, with the ability to be readily rehydrated prior to use [107]. At different pH levels, liposome systems show the ability to control phenolic release compared to non-liposomal extract [132]. Research should focus on the interactions between the liposomes and other constituents of the food and the ability of gut microbiota to metabolise them.

Polyphenols can exert prebiotic effects by promoting the growth of beneficial bacteria and suppressing the growth of harmful bacteria, thereby modulating gut microbiota composition and contributing to potential disease amelioration [133]. Therefore, the mechanism of interactions between the encapsulated polyphenols, gut microbiota and colon metabolism should be comprehensively studied. Encapsulation enhances the water solubility of polyphenols, protects them from gastric degradation, and enables their targeted release for gut microbiota modulation [134,135]. In Cassini et al. [132], liposomes made from Araucaria angustifolia extract enabled the controlled release and extended the intestinal residence time of phenolic compounds, enhancing their availability for microbiota-mediated activity. Food and nutraceutical products can be developed by applying these liposome stabilising systems with further studies mainly focusing on suitable phospholipids, and acid-labile and sensitive key targeted compounds. Due to the maintained stability throughout the conditions in the gastrointestinal tract and the selectivity in release, liposomes can be identified as one of the best strategies over other encapsulation methods, in terms of the optimum preservation of the bioactivity of the phenolics, until they reach the intestine.

### 4.5. Comparison Between the Encapsulation Methods

In food-related applications, microencapsulation through the coacervation technique offers several notable advantages over other encapsulation methods, including liposomes, spray-drying, solvent evaporation, ionic gelation, interfacial polymerisation, and molecular inclusion complexation. These benefits include exceptionally high encapsulation efficiency, operation under low or ambient temperatures, cost efficiency, and the absence of requirements for specialised equipment or toxic solvents [103]. In addition, complex coacervation offers several other benefits, including the requirement for only a low concentration of wall materials and suitability for controlled-release applications [104]. However, reduced solubility of proteins may lead to poor dispersion, weak electrostatic interactions, and instability of the resulting coacervates, thereby negatively affecting EE and the structural integrity, highlighting potential drawbacks. Since low solubility can restrict the application of proteins in aqueous food systems and may require additional processing modifications (incorporation of polymers), it is critical to monitor the physicochemical properties (pH value, ionic strength, biopolymer ratio, etc.) of the media for a stable coacervate.

Spray drying is a cost-effective and highly versatile technique that operates continuously under automated control. It is suitable for both heat-sensitive and heat-resistant materials, flexible in production capacity design, capable of controlling the particle size, and compatible with a wide range of wall materials [115,136,137]. Traditional spray-drying can be readily scaled up for industrial applications, enabling the production of kilograms to tons of dried materials. Another advantage is its ability to process solutions with higher viscosities [138]. However, during the spray-drying process, droplets with varying sizes are produced, which leads to a broader particle size distribution [136,139]. Such increases in particle size and heterogeneity can negatively affect the controlled release of phenolics. Therefore, selecting appropriate wall materials is essential to produce capsules with uniform particle sizes, high yield, and improved EE. Although the drying time is short, the loss of volatiles is a limitation of spray-drying, as the high processing temperatures can lead to an inevitable evaporation or degradation of volatile aromatics, flavours, and fragrances. In addition, despite the short exposure time, heat-sensitive bioactive compounds such as certain vitamins and proteins may still undergo thermal degradation during the process. Moreover, since spray-drying requires a designated spray-dryer with relevant equipment, the capital cost can be higher, highlighting a potential drawback at the initial operation stage.

Ionic gelation offers several advantages, including mild processing conditions that avoid the use of toxic organic solvents and high temperatures, making it suitable for encapsulating sensitive biological materials such as enzymes, proteins, and living cells [140,141]. The method is also considered safe and biocompatible because it commonly utilises food-grade, biodegradable, and non-toxic polymers like alginate and chitosan [136]. In addition, ionic gelation is cost-effective, as it requires only simple laboratory equipment rather than expensive and sophisticated machinery [141]. When polymer–drug interactions are properly optimised, the technique can also achieve high EE and loading capacity.

Despite its advantages, ionic gelation also has several limitations. Traditional ionic hydrogels often possess weak mechanical strength and may degrade rapidly when exposed to stress [140,142]. Conventional gelation techniques generally produce larger beads, which restricts their application in systems requiring nano-sized particles. Moreover, the difficulty in producing particles with a uniform size distribution is a drawback [141]. The relatively large pore size of hydrogel networks can lead to the leakage of smaller encapsulated compounds during storage or processing [143]. The technique is also less effective for highly water-soluble (hydrophilic) substances, frequently resulting in poor EE [140]. The stability of ionic gelation systems is also highly sensitive to environmental conditions, particularly pH and the presence of competing ions, which can weaken the electrostatic interactions responsible for capsule integrity.

Liposomes offer enhanced bioavailability due to their phospholipid bilayer structure, which closely resembles biological cell membranes. This structural similarity promotes improved absorption of phenolic compounds and facilitates their transport across biological barriers more efficiently. In addition, liposomes enable targeted and controlled release of encapsulated phenolics, allowing sustained or localised delivery that can enhance their functional, cosmetic, and therapeutic effectiveness. Liposomes can impart high biocompatibility, as they are commonly prepared using natural phospholipids, such as soy lecithin, which are biodegradable, non-toxic, and safe for food and pharmaceutical applications [144]. Furthermore, liposomal encapsulation can effectively mask the undesirable organoleptic characteristics of phenolic compounds, including bitterness in cynaropicrin in artichoke and astringency, thereby improving their acceptability in food products and oral formulations.

Conventional liposomes face several limitations related to stability, production, and large-scale application. They are thermodynamically unstable and are susceptible to aggregation, fusion, and leakage of encapsulated phenolic compounds during storage [145]. In addition, the phospholipids that form the liposomal bilayer can undergo oxidation and hydrolysis, leading to degradation of both the carrier system and the bioactive compounds [135,145]. Liposome production also presents challenges, as traditional preparation methods often result in low encapsulation efficiency and may involve toxic organic solvents that can damage phenolics [146]. Furthermore, plain liposomes exhibit limited resistance to the harsh conditions of the gastrointestinal tract, which may cause premature degradation before absorption [107]. Efficient encapsulation of plant phenolics is also difficult due to their intermediate polarity, which avoids easy association with either the aqueous core or lipid bilayer. Moreover, advanced techniques are required to produce stable and uniform nano-sized liposomes [146], which are often costly and difficult to scale up for industrial applications [147].

Out of these encapsulation methods, complex coacervation appears to be advantageous in terms of the EE. Mild processing conditions and suitability for protecting heat-sensitive phenolics make it highly attractive for food and pharmaceutical applications. Meanwhile, liposomes, on the other hand, offer excellent biocompatibility and enhanced bioavailability due to their phospholipid bilayer structure, which can effectively encapsulate both hydrophilic and hydrophobic phenolics. Overall, the selection between the ideal encapsulation strategy depends on the intended application, desired release characteristics, stability requirements, and economic feasibility of the encapsulation process.

## 5. Future Perspectives and Recommendations

The advancement of GEMs for valorising artichoke waste has demonstrated significant potential at the laboratory scale; nonetheless, translation to industrial applications remains a critical challenge. Future efforts must prioritise scaling-up and industrial feasibility by developing continuous and energy-efficient extraction systems that can be integrated into existing food processing infrastructures, overcoming their current drawbacks [69]. In this context, techno-economic analysis and LCA are essential to evaluate the process viability, ensuring that GEMs are not only environmentally sustainable but also economically competitive [148,149].

An integrated biorefinery is economically viable only when the prices of its products remain closely aligned with prevailing market values. From an environmental and engineering perspective, achieving a sustainable and acceptable biorefinery system requires the adoption of GEMs alongside efficient biochemical processing methods [148,149]. A promising direction lies in the integration of biorefinery and circular economy models, where artichoke waste is utilised as a multi-component resource rather than a single-value stream. Sequential extraction strategies could enable the recovery of polyphenols, pectin, dietary fibres (such as inulin) [150,151], and other bioactive compounds, followed by the conversion of residual biomass into bioenergy (cellulose and hemicellulose), paper pulp or biofertilisers [152,153]. Such utilisation can align with global sustainability goals and enhance resource efficiency while minimising waste generation.

To further improve extraction efficiency, the development of hybrid and intensified extraction technologies is expected to play a key role. The combination of techniques such as ultrasound, microwave, enzymatic treatment, and PEF may significantly enhance cell wall disruption and mass transfer, leading to higher yields and reduced processing times. Nevertheless, optimisation of these hybrid systems and the establishment of standardised operational parameters remain necessary to ensure reproducibility and scalability.

In parallel, the innovation of green solvents is gaining increasing attention, particularly the use of NADESs and other biobased solvent systems. These alternatives offer advantages in terms of sustainability and extraction efficiency; however, their widespread application requires further investigation into toxicological safety, allergenicity, regulatory acceptance, isolation of the compounds, and solvent recovery processes. The development of food-grade, recyclable solvent systems will be crucial for their adoption in the nutraceutical and food industries.

The successful translation of the extracted bioactives into commercial products represents another important objective. Artichoke waste extracts can be incorporated into functional foods [104,154], beverages [136,155], dietary supplements [28], and natural preservatives [137,156]. Table 3 shows several food applications of bioactives extracted from artichoke waste products, highlighting their potential nutritional benefits. Artichoke bioactive compounds could be used as a source of food supplements and health-promoting phenolic compounds, owing to their antioxidant, anticancer, and anticandidal activities, particularly for cancer patients undergoing chemotherapy and radiotherapy [138,157]. In Melini et al. [139,158], artichoke bracts contain approximately 15.43 g 100 g^−1^ DM of protein, and this level is notably higher than that of commonly used gluten-free ingredients such as rice flour (8.52 g 100 g^−1^ DM) [140,159] and corn flour (6.43 g 100 g^−1^) [141,160]. The protein content is comparable to that of emerging plant-based protein sources, including buckwheat flour (13.07 g 100 g^−1^ DM) [142,161], quinoa flour (15.7 g 100 g^−1^ DM) [143,162], and chickpea flour (17–22 g 100 g^−1^ DM) [139,163]. Melini et al. [139,158] further studied the suitability of powder made of external bracts in a flour mixture to prepare gluten-free rusks and found that the treated samples had higher protein, dietary fibre, phenolic and flavonoid contents. These findings suggest the potential applications of powder made of artichoke external bracts, substituting commercial flour types as a nutritious plant-based protein alternative. More studies should be focused on appropriate flour types and ratios, which can be mixed with artichoke bract powder to prepare a flour mixture that has enhanced functional, nutritional and physicochemical properties. Product development must further consider factors such as sensory attributes, formulation stability, consumer acceptance, and shelf-life. Collaboration between academia and industry will be key to bridging the gap between research and market-ready applications.

Beyond extraction, the stability, bioaccessibility, and bioavailability of recovered bioactive compounds must be thoroughly addressed. Many phenolic compounds are susceptible to degradation during processing and digestion, which can limit their functional efficacy. Advanced delivery systems, including nanoencapsulation and microencapsulation, offer promising strategies to enhance compound stability while enabling controlled release. Additionally, standardised in vitro digestion models and in vivo studies are needed to better understand how these compounds behave within the human gastrointestinal system.

Despite extensive in vitro evidence supporting the antioxidant and health-promoting properties of artichoke-derived compounds, clinical and nutritional validation remains limited. Future research should focus on well-designed animal studies and human clinical trials [28,138,144,157,163] to establish dose–response relationships, bioefficacy, and mechanisms of action, particularly in relation to chronic diseases such as diabetes, obesity, and cardiovascular disorders. Such validation is essential for substantiating health claims and supporting regulatory approval.

**Table 3:** Food products treated with artichoke by-products and their properties.

Food Matrix	Source of Artichoke Waste	Incorporation Method	Polyphenols and Antioxidant Activity	Functional and Nutritional Properties	Reference
Bakery products (Gluten-Free (GF) Rusks)	External bracts	Powder (substitution of 10% GF flour with powder)	Free phenolic compound content-138.99 mg GAE 100 g^−1^ d.w. (146.65% increment) Total flavonoid content-1123 mg CE 100 g^−1^ d.w (406.75% increment)	Protein–5.30 g 100 g^−1^ d.w. (33.17% increment) Dietary fibre–7.67 g 100 g^−1^ d.w. (101.31% increment) Inulin–0.83 g 100 g^−1^ d.w. (295.24% increment)	[139,158]
Bakery products (bread)	External bracts	Powder (substituting wheat flour with 10 g 100 g^−1^)	CGA-~ 26 mg 100 g^−1^ d.w 1,5 di-caffeoylquinic acids-~ 20 mg 100 g^−1^ d.w. 3,5 di-caffeoylquinic acids-~ 9 mg 100 g^−1^ d.w. Cellular antioxidant capacity (Caco–2 cell line) EC_50_ value-2.33 μg mL^−1^ ROS levels-from 40% to 70%	Protein–8.21 g 100 g^−1^ (13.40% increment) Fibre–6.6 g 100 g^−1^ (247.37% increment) Inulin–2.2 mg g^−1^ d.w. Glycaemic index 86.26 (2.98% reduction)	[145,164]
Bakery products (durum wheat bread)	External bracts	Powder (replacing re-milled durum wheat semolina at increasing levels (10 g 100 g^−1^)	Polyphenol content-0.57 mg GAE g^−1^ d.w. DPPH–0.55 mg TE g^−1^ d.w.	ND	[4]
	Stem		Polyphenol content-0.23 mg GAE g^−1^ d.w. DPPH–0.54 mg TE g^−1^ d.w.	ND	
	Mix of bracts and stem in 1:1 ratio		Polyphenol content-0.34 mg GAE g^−1^ d.w. DPPH–0.54 mg TE g^−1^ d.w.	ND	
Bakery products (Breadsticks)	External bracts	Powder (substituting 3% of flour)	TPC-354.68 mg GA 100 g^−1^ d.w. (30.24% increment) Flavonoids-18.64 mg CE 100 g^−1^ d.w (162.91% increment) DPPH-1.11 μmol TE g^−1^ d.w. (164.29% increment) ABTS-7.17 μmol TE g^−1^ d.w. (66.36% increment)	Protein 11.45 g 100 g^−1^ d.w. (7.14% reduction) Dietary fibre 6.42 mg 100 g^−1^ d.w. (28.4% increment, 154)	[104,154]
		Powder (substituting 5% of flour)	TPC-388.27 mg GA 100 g^−1^ d.w. (42.58% increment) Flavonoids-31.30 mg CE 100 g^−1^ d.w. (341.47% increment) DPPH-1.50 μmol TE g^−1^ d.w. (257.14% increment) ABTS-8.19 μmol TE g^−1^ d.w. (90.02% increment)	Protein 11.48 g 100 g^−1^ d.w. (6.89% reduction) Dietary fibre–7.59 mg 100 g^−1^ d.w. (51.8% increment)	
	Stem	Powder (substituting 3% of flour)	TPC-354.22 mg GA 100 g^−1^ d.w. (30.07% increment) Flavonoids-30.30 mg CE 100 g^−1^ d.w (327.36% increment) DPPH-1.54 μmol TE g^−1^ d.w. (266.67% increment) ABTS-7.91 μmol TE g^−1^ d.w. (83.53% increment)	Protein-11.34 g 100 g^−1^ d.w. (8.03% reduction) Dietary fibre-6.04 mg 100 g^−1^ d.w. (20.8% increment)	
		Powder (substituting 5% of flour)	TPC-420.01 mg GA 100 g^−1^ d.w. (54.23% increment) Flavonoids-42.35 mg CE 100 g^−1^ d.w. (497.32% increment) DPPH-2.66 μmol TE g^−1^ d.w. (533.33% increment) ABTS-9.24 μmol TE g^−1^ d.w. (114.39% increment)	Protein-11.25 g 100 g^−1^ d.w . (8.76% reduction) Dietary fibre-6.52 mg 100 g^−1^ d.w. (30.4% increment)	
Egg pasta	Mixture of stem and external bracts	10% of the extract (2 g powder in 40 mL of 80% ethanol) with 35% of semolina)	Uncooked pasta-TPC-2.05 mg GAE g^−1^ d.w. (10.22% increment)	Shelf life of fresh pasta stored at 5 °C was extended by ,^153^o	[152,153]
			Cooked pasta-TPC-0.73 mg GAE g^−1^ d.w. (35.19% increment)		
		10% of the extract (2 g powder in 40 mL of 80% ethanol) without semolina)	Uncooked pasta-TPC-2.04 mg GAE g^−1^ d.w. (6.25% increment)	Shelf life of fresh pasta stored at 5 °C was extended by two days	
			Cooked pasta-TPC-0.91 mg GAE g^−1^ d.w. (59.65% increment)		

d.w—dry weight, ROS–reactive oxygen species, ND—No data, TE—Trollox equivalent, CE—Catechin equivalents, GA—Gallic acid.

Finally, regulatory and safety considerations must not be overlooked. Comprehensive toxicological assessments, allergenicity evaluations, and adherence to food safety regulations are essential to ensure consumer protection. Moreover, the establishment of clear regulatory frameworks for waste-derived bioactives and standardised labelling practices will facilitate market entry and consumer trust in these sustainable products.

## 6. Conclusions

Globe artichoke waste, comprising stems, leaves, and bracts, constitutes a rich and sustainable source of bioactive compounds with significant nutritional value and health-promoting potential. The adoption of GEMs has demonstrated significant advantages over conventional methods by improving extraction efficiency while minimising solvent consumption, energy use, and environmental impact. The selection of GEMs and optimisation of extraction parameters can be applied to enrich artichoke by-product extracts with specifically targeted bioactive compounds. The diverse bioactivities of artichoke waste extracts, particularly their antioxidant and metabolic health benefits, support their suitability for functional food and nutraceutical applications. However, the susceptibility of phenolic compounds to degradation highlights the importance of encapsulation and stabilisation strategies, which have proven effective in enhancing bioavailability, storage stability, and controlled release. The incorporation of stabilised artichoke waste extracts into food systems not only improves product functionality but also aligns with sustainability and circular economic goals. Future research should focus on process scale-up, in vivo validation of health effects, and regulatory considerations to facilitate industrial implementation. Overall, the valorisation of globe artichoke waste through integrated green extraction, stabilisation, and food application strategies represents a promising pathway toward sustainable food innovation.

## Figures and Tables

**Table 2:** Encapsulation techniques used for polyphenols extracted from artichoke waste.

Technique	Part of the Plant	Process Conditions	Encapsulation Efficiency (EE) (%)	Antioxidant Activity and Other Important Factors	Reference
Nanoencapsulation (Polymeric nanoparticle using sputtering system)	Leaves	Temperature–67 °C Time–24 min Pressure drop–24.9 MPa	87	IC_50_-802 μg mL^−1^ Stored at 5 °C, After 14 days EE-84.6%, IC_50_-808 μg mL^−1^ After 28 days EE–83%, IC_50_-810 μg mL^−1^	[88]
Ionotropic gelation using a dropping method	Artichoke stem	Sodium alginate solution (2.25%) (pH = 5.5) Gel consolidation time–2 min Calcium chloride solution (2.50% (*w*/*v*)	ND	TPC loading efficiency–37.06%, Residual ABTS–25.97% Residual FRAP–20.90%	[102]
Chitosan-coated solid lipid nanoparticle (SLN) based double emulsion method (*w*/*o*/*w*)	Outer and external bracts	Mixing glycerol with monostearate, chloroform and sonicating with HCl to prepare w/o emulsion. The emulsion was mixed either with 2% *w/v* Poloxamer 407 or Tween 80 and then sonicated to prepare w/o/w emulsion. Mixing SLN with chitosan solution (0.5% *w*/*v*) in 1% *w/v* acetic acid	79.20 (with Poloxamer 407) 54 (with Tween 80)	ND	[106]
Solid lipid nanoparticle (SLN) based double emulsion method (*w*/*o*/*w*)	Outer and external bracts	Glycerol monostearate and chloroform were mixed and then sonicated with added HCl for 30 s to prepare w/o emulsion. Then, the emulsion was mixed either with 2% *w/v* Poloxamer 407 or Tween 80 and then sonicated for 30 s at 70% amplitude to prepare w/o/w emulsion. No coating with chitosan	74.00 (with Poloxamer 407) 57.00 (with Tween 80)	ND	
Spray drying	Artichoke leaf	Freeze-dried powdered ethanolic extract was dissolved in maltodextrin (dextrose equivalent of 7.5–9.9), or gum Arabic in a weight ratio of 1:5 (*w*/*w*). After mixing for 30 min, the samples were spray dried (inlet air temperature-150 °C, outlet-85 °C, feed rate-9 mL min^−1^)	90.21 (yield = 93.18%)	ND	[101]
Liposome entrapment	Leaves and bracts	Artichoke liposomes (Mixing soy lecithin (120 mg mL^−1^) and artichoke extract (20 mg mL^−1^), and hydrating with zein dispersion (2 mL of water) Artichoke zein liposomes (mixing soy lecithin (120 mg mL^−1^) and artichoke extract (20 mg mL^−1^), and hydrating with zein dispersion (2 mL containing 0.5 mg mL^−1^ of zein))	71.00 79.00	DPPH antioxidant activity (AA)–48.9 μg TE mL^−1^ ABTS AA–83.5 μg TE mL^−1^ CUPRAC AA–207.9 μg TE mL^−1^ FRAP AA–85.7 μg TE mL^−1^ After 2 h (pH 1.20)–68% CGA release After 6 h (pH 7.00)–14% CGA release DPPH AA–48.1 μg TE mL^−1^ ABTS AA–79.3 μg TE mL^−1^ CUPRAC AA–216.9 μg TE mL^−1^ FRAP AA–88.4 μg TE mL^−1^ After 2h (pH 1.20)–21% CGA release After 6 h (pH 7.00)–34% CGA release	[107]
Ionic gelation (Chitosan nanoparticles (CN))	Chlorogenic acid (CGA)	Mixing sodium tripolyphosphate and chitosan to prepare CN. Then mixing CN with CGA. Concentration of CGA used in CN- 2 µM	74.43	CGA Loading (CGA loaded per unit weight of CN)–1.49 µM	[108]
		Concentration of CGA used in CN- 10 µM	62.30	CGA Loading–6.23 µM	
		Concentration of CGA used in CN- 20 µM	60.21	CGA Loading–12.04 µM	

AA—Antioxidant activity, TE—Trollox equivalent, ND—No data.

## Data Availability

No new data were created or analyzed in this study. Data sharing is not applicable to this article.

## Abbreviations

The following abbreviations are used in this manuscript:

EP	Edible portion
CGA	Chlorogenic acid
CQA	Caffeoylquinic acid
DPPH	2,2-diphenyl-1-picrylhydrazyl
CUPRAC	Cupric-ion-reducing antioxidant capacity
ABTS	2,2′-azinobis(3-ethylbenzothiazoline-6-sulfonic acid)
FRAP	Ferric Reducing Antioxidant Power
TE	Trollox equivalent
CE	Catechin equivalent
TPC	Total polyphenolic content
GAE	Gallic acid equivalent
fw	Fresh weight
ROS	Reactive oxygen species
DM	Dry matter
LOOH	Lipid hydroperoxide
FFAs	Free fatty acids
AFS	Artichoke floral stem
CAT	Catalase
GPx	Glutathione peroxidase
SOD	Superoxide dismutase
RAI	Redox activity inhibition
TFC	Total flavonoid content
MAE	Microwave-assisted extraction
NADES	Natural deep eutectic solvent
SLE	Solid–liquid extraction
REACH	Registration, evaluation, authorisation and restriction of chemicals
GEM	Green extraction methods
DESE	Deep eutectic solvent extraction
HPAE	High-pressure assisted extraction
UAE	Ultrasound-assisted extraction
PEF	Pulsed electric field
PEFE	Pulsed electric field extraction
EAE	Enzyme-assisted extraction
SWE	Subcritical water extraction
CGAE	Chlorogenic acid equivalent
d.w	Dry weight
d.e	Dry extract
HBD	Hydrogen bond donor
HBA	Hydrogen bond acceptor
ND	No data
EtOH	Ethanol
TEAC	Trolox equivalent antioxidant capacity
QE	Quercetin equivalent
EUMAE	Hybrid ultrasound–microwave-assisted extraction
EUAE	Ultrasound-assisted extraction
EMAE	Enzymatic pre-treatment combined with microwave-assisted extraction
SCO_2_E	Supercritical carbon dioxide extraction
SCF	Supercritical fluid
EE	Encapsulation efficiency
AA	Antioxidant activity
SLN	Solid lipid nanoparticle
CN	Chitosan nanoparticles
GF	Gluten free
SP	Soy protein
